# High‐Current‐Density Acidic CO_2_ Electroreduction to Formic Acid Enabled by Multiscale Microenvironment Regulation Over Defect‐Rich Sn/SnO_x_

**DOI:** 10.1002/advs.77692

**Published:** 2026-09-08

**Authors:** Qiaoqi Guo, Bowen Jiang, Le Huang, Kunyu Jiang, Rongrong Tang, Qinong Zhu, Huajun Feng, Mengyang Fan, Yijing Xia

**Affiliations:** ^1^ Zhejiang Key Laboratory of Solid Waste Pollution Control and Resource Utilization School of Environmental Science and Engineering Zhejiang Gongshang University Hangzhou People's Republic of China; ^2^ College of Environment and Resources College of Carbon Neutral Zhejiang A&F University Hangzhou Zhejiang People's Republic of China; ^3^ Jinhua Academy of Zhejiang Chinese Medical University Jinhua Zhejiang People's Republic of China; ^4^ College of Carbon Neutrality Future Technology Sichuan University Chengdu People's Republic of China

**Keywords:** acidic CO_2_ electroreduction, formic acid, high current density, janus gas‐diffusion electrode, Sn/SnO_x_ nanoclusters

## Abstract

Acidic CO_2_ electrolysis to formic acid circumvents carbonate formation and enables direct product recovery, yet is hindered by severe competition between formate‐producing *OCHO and hydrogen‐producing *H intermediates. Here, we develop a hierarchical site‐solvation‐transport regulation strategy to direct this competition toward formic acid. Mixed, undercoordinated Sn/SnO_x_ nanoclusters provide catalytic environments associated with the *OCHO pathway, while K^+^‐dependent interfacial solvation is associated with reduced hydrogen evolution reaction (HER) competition and enhanced formic acid selectivity. A Janus asymmetric‐wettability electrode further stabilizes CO_2_ mass transport and prevents electrolyte flooding, sustaining a favorable cathodic microenvironment. This integrated strategy achieves ∼85% formic acid Faradaic efficiency in acidic media with an initial 18 h stability window at 400 mA cm^−2^. This work establishes a multiscale strategy for coupling intermediate selectivity with mass‐transport management in acidic CO_2_ electrolysis.

## Graphical abstract

Acidic CO_2_ electroreduction to formic acid is enabled by integrating mixed Sn/SnO_x_ nanoclusters, K^+^‐regulated interfacial solvation, and Janus wettability. Coordinated control of active sites, interfacial water, and gas‐liquid transport suppresses hydrogen evolution and delivers approximately 85% HCOOH Faradaic efficiency at 400 mA cm^−2^.

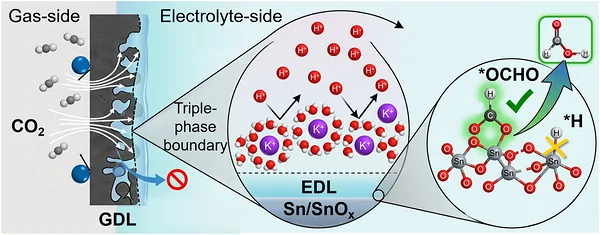

## 1 Introduction

Electrochemical CO_2_ reduction (CO_2_RR) offers a sustainable route to convert renewable electricity and CO_2_ into value‐added chemicals and fuels, with catalyst composition, intermediate binding, and the local reaction environment jointly governing the resulting product distribution [1, 2, 3, 4, 5]. Acidic electrolysis offers a distinctive advantage specifically for formic acid production. It mitigates the substantial carbonate crossover issue prevalent in neutral and alkaline systems [6, 7, 8], but crucially, it enables the direct generation of predominantly protonated formic acid in an acidic electrolyte (HCOOH). This direct pathway circumvents the energy‐intensive downstream acidification and separation steps typically required to recover liquid fuel from formate salts [9, 10, 11], making acidic operation attractive for reactor architectures designed for direct formic acid production. Previous reviews have established the typical CO_2_ electroreduction pathways, applicable catalyst types, and performance thresholds [12]. Formic acid/formate selectivity and reaction conditions are highly pH‐dependent, with neutral/alkaline electrolytes yielding HCOO^−^ and inhibiting hydrogen evolution yet inducing carbonate byproducts and CO_2_ loss, while acidic systems generate HCOOH and eliminate post‐acidification but suffer HER [13].

Direct HCOOH synthesis thus demands coordinated regulation of catalyst selectivity, proton migration, and CO_2_ mass transport. Direct CO_2_‐to‐formic acid electrosynthesis has been explored in several reactor configurations, each offering a different balance among catalyst evaluation, reaction rate, and product recovery. Conventional H‐type cells provide well‐defined liquid‐phase conditions for assessing catalyst behavior, but the low solubility of CO_2_ in aqueous electrolytes and its long diffusion distance to the electrode limit the attainable current density and formic acid productivity [9, 14]. Three‐compartment solid‐electrolyte reactors instead separate the cathode and anode chambers from a central product‐collection compartment. Cathodically generated formate and protons transported from the anode side combine in the central compartment, enabling continuous collection of electrolyte‐free or concentrated HCOOH solutions [10, 15, 16, 17]. Although this architecture reduces the need for downstream acidification and salt removal, the additional membranes and solid‐electrolyte layer increase ohmic resistance, complicate ion and water management, and add to device complexity. Zero‐gap membrane‐electrode assemblies shorten the ionic transport distance and offer a compact platform for high‐rate CO_2_ electrolysis [7, 9]. Their performance, however, remains sensitive to membrane hydration, pressure balance, product crossover, salt accumulation, and catalyst‐layer flooding. The compressed configuration also limits direct access to the cathode/electrolyte interface for mechanistic investigation.

Gas‐diffusion‐electrode flow cells offer a complementary platform for high‐rate CO_2_ reduction under controlled acidic conditions. Gaseous CO_2_ is supplied through a porous diffusion layer, substantially shortening the transport distance relative to dissolved‐CO_2_‐fed H‐cells. Meanwhile, a circulating catholyte allows the pH, electrolyte composition, and cation environment to be independently adjusted [6, 18]. This configuration is therefore well suited to investigating the combined effects of catalyst structure and interfacial environment at high current densities. At pH 2, the liquid product exists predominantly as protonated HCOOH rather than HCOO^−^. Nevertheless, the gas‐liquid‐solid interface is highly sensitive to electrolyte intrusion, catalyst‐layer wetting, and local CO_2_ depletion. Gas‐diffusion‐electrode flow cells thus provide a platform for examining the coupled roles of active‐site chemistry, interfacial solvation, and electrode‐scale mass transport. However, they do not inherently solve the problems of product dilution, electrolyte flooding, membrane crossover, or long‐term interface instability [17]. In this work, the flow cell is therefore used primarily to study acidic CO_2_ reduction at high current density.

Despite these reactor‐level advantages, acidic operation presents a selectivity challenge at the cathode. Formic acid formation and HER compete through the adsorption of CO_2_‐derived intermediates and *H, respectively. It depends on the adsorption properties of the electrode, pH, applied potential, CO_2_ partial pressure, and local mass transport [13]. In proton‐rich media, rapid *H formation can occupy active sites before oxygen‐bound CO_2_ intermediates are stabilized. Electrolyte flooding or insufficient CO_2_ supply further lowers the local CO_2_‐to‐proton ratio and promotes HER [19, 20]. Selective HCOOH production at high current density therefore requires coordinated regulation of intermediate adsorption, interfacial proton transfer, and gas‐liquid transport [21, 22, 23].

Several catalyst families can reduce CO_2_ to formate or formic acid, including molecular complexes and materials based on Sn, Bi, In, Pb, and Pd [12]. Among them, Bi‐based catalysts have attracted considerable attention because weak hydrogen adsorption suppresses HER, while their affinity for oxygen‐bound intermediates favors formate formation. Low‐dimensional bismuthene exposes abundant undercoordinated sites and can achieve high formate selectivity at relatively moderate cathodic potentials [24]. Regulation of the local environment of Bi nanosheets has also enabled selective HCOOH production in strongly acidic media [25]. Bi‐based bromide perovskites have shown similar promise through the formation of active Bi‐containing phases under electrochemical conditions [26]. These results establish Bi as an important benchmark in terms of selectivity and operating potential.

Sn was selected in this work because it offers a practical and structurally tunable platform for studying formic acid formation under acidic conditions. Sn‐based surfaces generally favor oxygen‐bound intermediates over strongly adsorbed *CO, making the *OCHO pathway accessible [12, 27]. Computational and experimental studies have placed Sn near the favorable region of the activity relationship between *OCHO binding and formate production [27]. Sn is also a non‐noble metal with relatively low cost and low toxicity compared with Pb. These properties make it suitable for integration into carbon‐supported gas‐diffusion electrodes [14, 28, 29, 30, 31].

A further advantage of Sn is that its catalytic properties can be adjusted through particle size, oxidation state, defects, support interactions, and metal‐oxide interfaces [27]. Their surfaces generally bind oxygenated intermediates more favorably than *CO, which makes the *OCHO pathway accessible for formic acid formation [32]. Oxidized Sn species, oxide‐derived Sn, and Sn/SnO_x_ interfacial motifs have been associated with enhanced stabilization of *OCHO and improved selectivity toward formic acid [33]. Defects and local coordination asymmetry can further tune adsorption energetics and modify proton‐coupled electron‐transfer steps [16]. However, in acidic media, the proton‐rich environment drastically alters the interfacial reaction landscape. The abundant local proton supply kinetically accelerates the adsorption of *H, which vigorously competes with CO_2_ activation for the limited active sites [34]. Even catalysts with a strong intrinsic affinity for *OCHO often suffer a severe decline in carbon product selectivity, because the competitive *H formation outpaces *OCHO stabilization, ultimately diverting the cathodic current toward HER [25, 35]. Previous studies have mainly regulated the catalyst, interfacial environment, or electrode structure separately [22, 36, 37, 38, 39, 40, 41, 42, 43, 44, 45, 46, 47, 48, 49].

Here, we integrate these three levels of control for acidic CO_2_ electrolysis. Defect‐rich Sn/SnO_x_ nanoclusters favor the *OCHO pathway, K^+^‐associated interfacial regulation shifts the competition from *H toward *OCHO, and a Janus electrode limits excessive electrolyte intrusion while maintaining CO_2_ transport. This site‐solvation‐transport strategy enables selective HCOOH production at high current density and extends catalyst design to the reaction interface.

## 2 Experimental Section

### 2.1 Chemicals and Materials

Citric acid sodium salt dihydrate or trisodium citrate dihydrate, potassium sulfate, potassium bicarbonate, potassium hydroxide, lithium sulfate, sodium sulfate, cesium sulfate, tin chloride dihydrate, and sodium borohydride were purchased from Shanghai Macklin Biochemical Co., Ltd. Carbon black VXC‐72 was purchased from Cabot Corporation. All reagents were of analytical grade and used without further purification. Ultrapure water with a resistivity of at least 18 MΩ cm was used throughout the experiments.

### 2.2 Synthesis of Sn/SnO_x_@CB Catalysts

Sn/SnO_x_@CB catalysts were synthesized by a wet‐chemical reduction method. In a typical procedure, predetermined amounts of SnCl_2_·2H_2_O and trisodium citrate dihydrate were dissolved in 100 mL of ultrapure water under magnetic stirring. Carbon black was then added, and the suspension was ultrasonicated to obtain a homogeneous dispersion. A NaBH_4_ solution was slowly injected into the suspension, followed by vigorous stirring at 25°C. After the reaction, the suspension was centrifuged at 100 00 rpm for 5 min. The precipitate was washed repeatedly with ultrapure water and centrifuged. The final product was dried in a vacuum oven to obtain Sn/SnO_x_@CB catalysts with different Sn contents.

The precursor amounts used for the catalyst series were as follows: CB, SnCl_2_·2H_2_O 0 g, trisodium citrate dihydrate 1.1764 g, NaBH_4_ 0.1 m, carbon black 0.2 g; Sn/SnO_x_‐1@CB, SnCl_2_·2H_2_O 0.0235 g; Sn/SnO_x_‐2@CB, SnCl_2_·2H_2_O 0.0470 g; Sn/SnO_x_‐4@CB, SnCl_2_·2H_2_O 0.0959 g; Sn/SnO_x_‐8@CB, SnCl_2_·2H_2_O 0.1880 g; and Sn/SnO_x_‐16@CB, SnCl_2_·2H_2_O 0.3760 g. For all Sn‐containing samples, the amounts of trisodium citrate dihydrate, NaBH_4_, and carbon black were kept constant.

### 2.3 Electrode Preparation

Catalyst inks were prepared by dispersing the catalyst powder in a mixed solvent of ethanol, deionized water, and 5 wt.% Nafion solution with a volume ratio of 3:1:0.01. The dispersion was ultrasonicated to obtain a homogeneous ink with a catalyst concentration of 5.0 mg mL^−1^. The ink was drop‐cast onto a gas‐diffusion electrode with a catalyst loading of 0.75 mg cm^−2^. The prepared electrodes were dried overnight before electrochemical measurements. The resulting electrodes were denoted CB, Sn/SnO_x_‐1@CB, Sn/SnO_x_‐2@CB, Sn/SnO_x_‐4@CB, Sn/SnO_x_‐8@CB, and Sn/SnO_x_‐16@CB.

### 2.4 Preparation of Substrates With Different Wettability

The DH, DSH, and Janus substrates were prepared from the same PTFE‐free carbon paper. The untreated carbon paper was used as the DH substrate. The DH denotes the untreated, relatively more wettable substrate used as the double‐wettable reference. The DSH substrate was prepared by airbrushing a 5 wt.% PTFE dispersion onto both sides of the carbon paper at a PTFE loading of 0.75 mg cm^−2^ per side. The Janus substrate was prepared by applying the same PTFE dispersion exclusively to the gas‐facing side at a loading of 0.75 mg cm^−2^, while the opposite side was masked during coating. The PTFE‐treated substrates were dried at 80°C for 30 min and thermally treated at 340°C for 30 min under N_2_ [43, 50]. The catalyst ink was subsequently deposited on the catholyte‐facing side at a catalyst loading of 0.75 mg cm^−2^.

### 2.5 Characterization

Scanning electron microscopy (SEM) and energy‐dispersive x‐ray spectroscopy (EDS) were used to characterize morphology and elemental distribution. Transmission electron microscopy and high‐resolution (TEM) were used to examine nanostructure and particle size. x‐ray diffraction was used to identify crystalline phases. X‐ray photoelectron spectroscopy (XPS) was used to analyze surface chemical states. X‐ray absorption spectroscopy was collected at the Sn K‐edge. Data were analyzed using the Athena and Artemis modules in the Demeter software package. Contact angles were measured at room temperature using liquid droplets of 10 µL. Gas contact angles were measured by immersing the electrode in ultrapure water and introducing a gas bubble of 15 µL from below the electrode. The captive‐bubble angle was defined through the aqueous phase outside the bubble. All post‐electrolysis electrodes were gently rinsed with ultrapure water and dried at 60°C before measurement. Linear sweep voltammetry was performed in a three‐electrode configuration using catalyst‐coated carbon paper as the working electrode, Ag/AgCl as the reference electrode, and Pt wire as the counter electrode. The potential was swept from 0 to −1.71 V at a scan rate of 10 mV s^−1^. Before measurements, the electrolyte was purged with CO_2_ or Ar for at least 30 min. Electrochemical impedance spectroscopy was measured using an AC impedance program over a frequency range from 10^5^ to 0.01 Hz.

### 2.6 Electron Paramagnetic Resonance (EPR) Measurements

Continuous‐wave electron paramagnetic resonance (CW‐EPR) spectra were recorded at room temperature (295 K) using a Bruker EMXplus‐6/1 spectrometer operating in the X‐band. The microwave frequency was approximately 9.84 GHz (9.835–9.844 GHz), and the microwave power was set to 3.17 mW with an attenuation of 18 dB. The magnetic field was swept from 3455 to 3555 G, with a center field of 3505 G and a sweep width of 100 G. Field modulation was applied at 100 kHz with a modulation amplitude of 3.0 G. Each spectrum was collected over 1000 data points with a sweep time of 30 s, a conversion time of 30 ms, a time constant of 0.01 ms, and a receiver gain of 30 dB. Three consecutive scans were accumulated to improve the signal‐to‐noise ratio. The spectra were baseline‐corrected using the Bruker data‐processing software. The corresponding *g* values were determined from the resonance field and the measured microwave frequency according to *g* = ^hν^ /μ_B_B_res_ where h, ν, μ_*B*_, *B_res_* denote Planck's constant, the microwave frequency, the Bohr magneton, and the resonance magnetic field, respectively.

### 2.7 In Situ ATR‐FTIR Spectroscopy

In situ attenuated total reflection Fourier‐transform infrared spectroscopy was performed on a Thermo Scientific Nicolet iS50 spectrometer equipped with a liquid‐nitrogen‐cooled MCT detector. Measurements were carried out in a sealed cell to minimize interference from atmospheric CO_2_. The FTIR chamber was purged with Ar for at least 90 min before experiments to reduce residual CO_2_ and water vapor in the optical path. A catalyst‐modified silicon crystal was used as the working electrode, Ag/AgCl as the reference electrode, and Pt wire as the counter electrode. All spectra were collected in CO_2_‐saturated 0.5 m K_2_SO_4_ adjusted to pH 2. Chronoamperometric measurements were performed over the potential range from open‐circuit potential to −2.0 V vs. RHE.

### 2.8 Operando Raman Spectroscopy

Operando Raman measurements were carried out in a custom electrochemical cell using a three‐electrode configuration. Catalyst‐loaded carbon paper was used as the working electrode, Pt wire as the counter electrode, and Ag/AgCl as the reference electrode. A silicon wafer was used for spectral calibration. Raman spectra were collected under constant potential from −0.6 to −2.0 V vs. Ag/AgCl. A 532 nm laser with a power of 1.05 mW was used, and spectra were collected over the range of 2000–4000 cm^−1^.

### 2.9 CO_2_ Electroreduction Measurements

CO_2_ electroreduction was performed in a three‐electrode flow cell. The catalyst‐coated gas‐diffusion electrode with a geometric area of 1 cm^2^ was used as the working electrode. Ag/AgCl saturated with KCl was used as the reference electrode, and a Pt plate was used as the counter electrode. The cathode catalyst layer faced the electrolyte chamber, while the gas chamber was separated from the catholyte by the working electrode. The cathode and anode compartments were separated by a proton‐exchange membrane. Unless otherwise specified, 0.5 m K_2_SO_4_ adjusted to pH 2 with H_2_SO_4_ was used as the electrolyte. The catholyte and anolyte were circulated by a dual‐pump system. CO_2_ was fed into the gas chamber at 30 sccm using a mass‐flow controller. All electrochemical measurements were performed using a CHI 660 electrochemical workstation. Although the potentials were recorded against the Ag/AgCl reference electrode during measurement, all electrode potentials reported in this work were converted to the reversible hydrogen electrode (RHE) scale according to:

$$E_{RHE}=E_{Ag/AgCl}+{E^{0}}_{Ag/AgCl}+0.0591\times pH$$

where E_Ag/AgCl_ is the measured potential vs. the Ag/AgCl reference electrode, E^0^_Ag/AgCl_ is the standard potential of the Ag/AgCl reference electrode saturated with KCl, and pH is the measured electrolyte pH. For the saturated KCl Ag/AgCl reference electrode used here, E^0^_Ag/AgCl_ was taken as 0.197 V at 25°C.

### 2.10 Product Analysis and Faradaic Efficiency Calculation

Gaseous products (H_2_ and CO) were analyzed by gas chromatography (GC7920, Beijing China Education Au‐light Co., Ltd.) equipped with a series thermal conductivity detector (TCD) and flame ionization detector (FID). A Porapak Q packed column (80/100 mesh, 2 m × 3 mm) coupled with a 5A molecular sieve column (60/80 mesh, 1 m × 3 mm) was used for separation. High‐purity nitrogen (99.999%) served as carrier gas (30 mL·min^−1^), with column oven temperature programmed as: 40°C for 5 min, ramp to 150°C at 10°C·min^−1^, and hold for 10 min. TCD detected H_2_; FID equipped with a methanizer quantified CO, and external standard calibration with known concentration gas mixtures was used for quantification.

The liquid product was quantified by ion chromatography (Dionex ICS‐5000+) via external calibration as formate and reported as the HCOOH equivalent. Separation was performed on an IonPac AS18 analytical column (4 × 250 mm) with an IonPac AG18 guard column (4 × 50 mm), using 20 mm KOH as eluent (1.0 mL·min^−1^) at 30°C. The injection volume was 25 µL, and detection was conducted with a conductivity detector. A calibration curve (0.1–10 mm HCOOH standards) was established to calculate sample concentration based on peak area comparison. H_2_ and formic acid were quantified from calibrated response factors.

The Faradaic efficiency was calculated according to

$$\mathrm{FE}=\frac{\mathrm{znF}}{Q}\times 100\%$$

where *z* is the number of electrons transferred, *n* is the amount of product, *F* is the Faraday constant, and *Q* is the total charge passed during electrolysis.

The CO_2_ molar inlet flow rate was calculated from the volumetric flow rate according to

$$v_{\mathrm{in}}=\frac{F_{\mathrm{in}}}{V_{m}\times 60}$$

where v is the molar flow rate of CO_2_, I is the volumetric flow rate in mL min^−1^, and V is the molar volume of gas at 25°C and 1 atm, 24.45 L mol^−1^.

The molar production rate of formic acid was calculated from

$$v_{\mathrm{HCOOH}}=\frac{I\times FE_{\mathrm{HCOOH}}}{\mathrm{zF}}$$

where *I* is the applied current, FE is the Faradaic efficiency for formic acid, *z* = 2, and *F* = 96485 C mol^−1^. The single‐pass carbon efficiency was calculated based on the stoichiometric one‐to‐one conversion of CO_2_ to formic acid.

The HCOOH partial current density was calculated as:

$$j_{\mathrm{HCOOH}}=j_{\mathrm{total}}\times FE_{\mathrm{HCOOH}}$$

The area‐normalized HCOOH production rate was calculated using:

$$r_{\mathrm{HCOOH}}=\frac{j_{\mathrm{HCOOH}}}{2F}$$

The electrolyzer‐level specific electrical energy consumption was calculated from:

$$\mathrm{SE}\;C_{\mathrm{HCOOH}}=\frac{V_{\mathrm{cell}}\times j_{\mathrm{total}}}{m_{\mathrm{HCOOH}}}$$

where V_cell_ is the measured cell voltage, and *m_HCOOH_* is the area‐normalized mass production rate of HCOOH. The reported energy consumption includes only the direct‐current electrical input to the electrolyzer. The cathode/anode electrolyzer DC electricity consumption is based on the measured full‐cell voltage

### 2.11 Density Functional Theory Calculations

Density functional theory calculations were performed using the Vienna Ab initio Simulation Package. The exchange‐correlation potential was described using the generalized gradient approximation in the Perdew–Burke–Ernzerhof form. The projector augmented‐wave method was used to describe ion core‐valence electron interactions. The plane‐wave cutoff energy was set to 450 eV. Structural models were relaxed until the Hellmann‐Feynman forces were less than 0.02 eV Å^−1^ and the energy change was less than 10^−5^ eV. Dispersion interactions were described using Grimme's DFT‐D3 correction. Gamma‐centered k‐point sampling of 2 × 2 × 1 was used. A vacuum space of 13 Å was applied along the *z* direction.

A simplified carbon‐supported Sn model was used to compare the influence of different alkali‐metal species on the adsorption of key reaction intermediates. The model consisted of a carbon substrate supporting a Sn motif.

For comparison of alkali‐metal effects, Li, Na, K, or Cs species were introduced near the Sn‐adsorbate region, and the structures were geometrically optimized under the same computational settings. The theoretical models are used to compare relative structural, electronic, and energetic trends within the same simplified Sn/C framework.

The Gibbs free‐energy change was calculated as

ΔG=ΔE+ΔZPE−TΔS

where Δ*E* is the electronic energy obtained from DFT, Δ*ZPE* is the zero‐point‐energy correction, *T* is the temperature, and Δ*S* is the entropy change. A more negative adsorption free energy indicates stronger adsorption under the adopted computational reference conditions.

### 2.12 Prospective Life‐Cycle and Techno‐Economic Assessment

A prospective cradle‐to‐gate screening life‐cycle assessment and techno‐economic assessment were conducted to evaluate the environmental and economic implications of acidic CO_2_ electroreduction to formic acid over the Sn/SnO_x_‐4@CB catalyst. The functional unit was defined as 1 kg of pure HCOOH. The system boundary included catalyst and electrode preparation, CO_2_ supply and recycling, electrolysis, auxiliary electricity consumption, product separation, electrolyte handling, and waste treatment. The manufacture of the electrolyzer, pumps, piping, and other balance‐of‐plant equipment was excluded from the base‐case LCA because reliable material inventories and service‐life data were not available. These components were considered in the TEA through an installed electrolyzer cost.

The electrolysis electricity demand was calculated from the theoretical two‐electron charge requirement for CO_2_‐to‐HCOOH conversion:

$$E_{\mathrm{elec}}=\frac{1.164V_{\mathrm{cell}}}{FE_{\mathrm{HCOOH}}}$$

where 1.164 kWh kg^−1^ is the theoretical electricity requirement at 100% efficiency. The experimentally demonstrated values at 400 mA cm^−2^, namely an HCOOH Faradaic efficiency of approximately 85% and a cell voltage of approximately 7.4 V, were used in the base case. Auxiliary electricity consumption was assumed to be 10% of the electrolysis demand. Because the HCOOH outlet concentration was not determined in the present study, a separation energy of 5.0 kWh kg^−1^ HCOOH was used as a screening‐level base case, with a sensitivity range of 0.5–8.0 kWh kg^−1^.

The stoichiometric CO_2_ requirement was 0.956 kg CO_2_ kg^−1^ HCOOH. Unreacted CO_2_ was assumed to be recycled in the base case. A CO_2_ supply factor of 0.20 kg CO_2_e kg^−1^ CO_2_ and a grid electricity carbon intensity of 0.55 kg CO_2_e kWh^−1^ were used. The catalyst contribution was calculated from the measured catalyst loading of 0.75 mg cm^−2^, the HCOOH production rate at 400 mA cm^−2^, the 85% Faradaic efficiency, and the demonstrated 18 h stability period. A conservative catalyst synthesis factor of 50 kg CO_2_e kg^−1^ catalyst was applied [51, 52].

For the TEA, the production capacity was set to 10 000 t HCOOH yr^−1^ with 7884 operating hours per year. The economic boundary included electricity, CO_2_ supply, catalyst, membrane and gas‐diffusion electrode replacement, electrolyzer capital cost, fixed operation and maintenance, electrolyte handling, and waste treatment. The estimated production cost was calculated on a mass basis and used to compare the current experimental case with prospective improved operating scenarios [53, 54].

## 3 Results and Discussion

### 3.1 Construction and Structural Identification of Defect‐Rich Sn/SnO_x_ Nanoclusters

A series of carbon‐black‐supported Sn catalysts was prepared by varying the amount of Sn precursor and denoted Sn/SnO_x_‐1@CB, Sn/SnO_x_‐2@CB, Sn/SnO_x_‐4@CB, Sn/SnO_x_‐8@CB, and Sn/SnO_x_‐16@CB. Scanning electron microscopy and elemental mapping showed that C, O, and Sn were distributed throughout the conductive carbon framework without obvious microscale phase segregation (Figure S1). This relatively uniform dispersion minimizes the influence of macroscopic compositional inhomogeneity and allows the effects of Sn loading and nanocluster size to be systematically evaluated.

**FIGURE 1:**
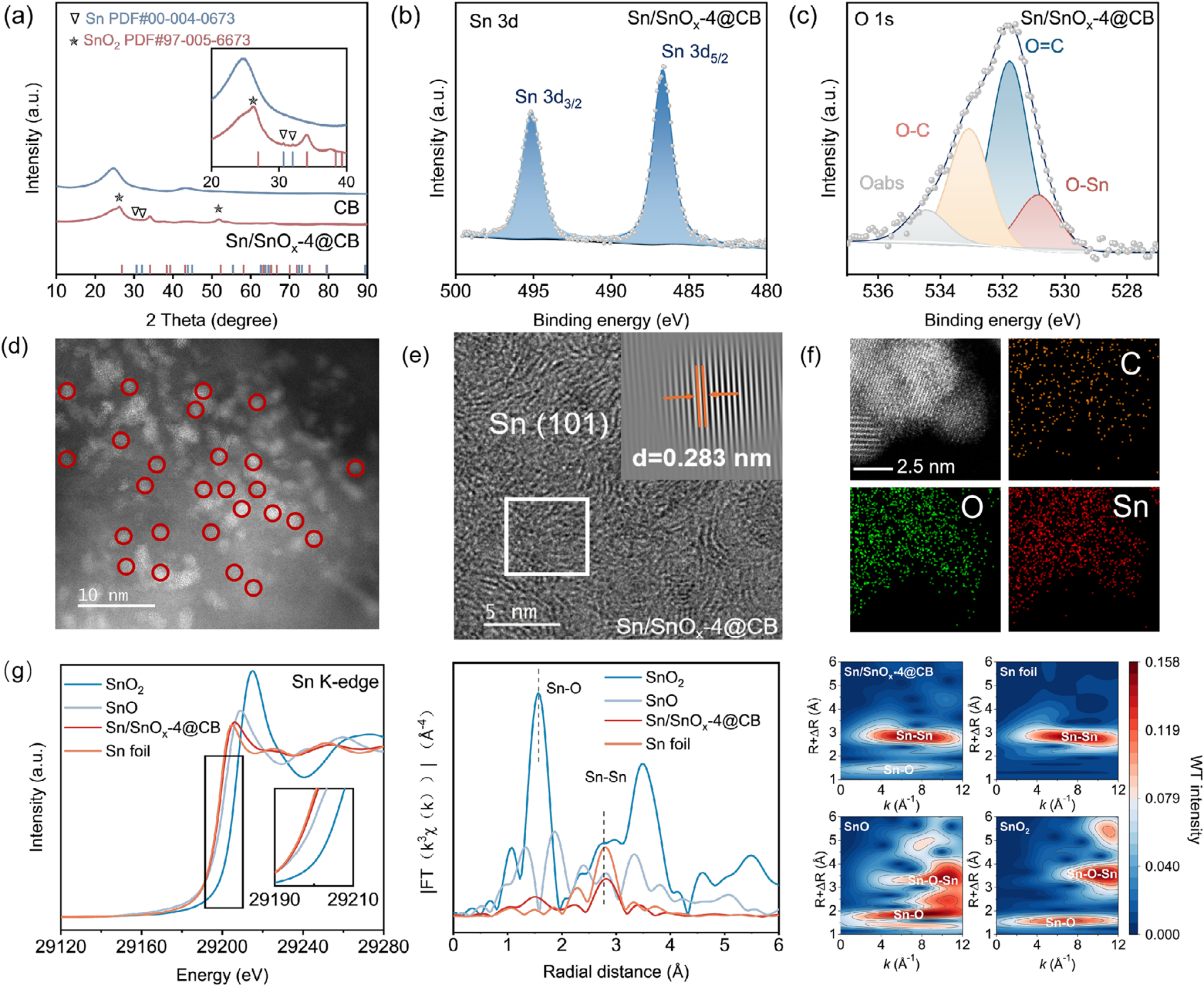
Structural characterization of Sn/SnO_x_‐4@CB. (a) XRD patterns of CB and Sn/SnO_x_‐4@CB catalysts. (b) High‐resolution Sn 3d XPS spectrum of Sn/SnO_x_‐4@CB. (c) High‐resolution O 1s XPS spectrum of Sn/SnO_x_‐4@CB. (d) Representative TEM or HAADF‐STEM image of Sn/SnO_x_‐4@CB. (e) HRTEM image of Sn/SnO_x_‐4@CB. (f) Elemental mapping or line‐scan profile of Sn/SnO_x_‐4@CB. (g) Sn K‐edge XANES spectra, Fourier‐transformed EXAFS spectra, and wavelet‐transform EXAFS maps of Sn/SnO_x_‐4@CB compared with Sn foil and SnO_2_.

The crystalline phases were first examined by x‐ray diffraction. As shown in Figure 1a, the diffraction pattern of Sn/SnO_x_‐4@CB contains SnO_2_‐related features, including reflections at approximately 27° and 34°, consistent with the (110) and (101) planes of SnO_2_ (PDF#97‐005‐6673), respectively. Weaker reflections attributable to metallic Sn (PDF#00‐004‐0673) are also observed, indicating the coexistence of oxidized and metallic crystalline domains. Because XRD probes long‐range order across the powder ensemble, a relatively small amount of crystalline Sn can still produce detectable diffraction features. The Sn‐related reflections generally became more pronounced with increasing Sn precursor amount across the catalyst series (Figure S2).

XPS was subsequently used to examine the near‐surface chemical states and Sn‐O bonding environment. The survey spectra confirmed the presence of Sn after loading onto the carbon support (Figure S3a). The high‐resolution Sn 3d spectrum of Sn /SnO_x_‐4@CB shows the characteristic Sn 3d_5/2_ and Sn 3d_3/2_ doublet (Figures 1b and S3b). The spectrum is dominated by oxidized Sn species and detects a minor Sn contribution. Moreover, the overlapping Sn 3d binding‐energy ranges of Sn^2+^ and Sn^4+^ preclude their unambiguous quantitative separation. The O 1s spectrum was deconvoluted into O‐C, O═C, and O‐Sn components (Figures 1c and S4), with the O‐Sn contribution further supporting the presence of oxidized Sn species. These results indicate that oxidized Sn dominates the XPS‐probed region but do not exclude the minor metallic Sn component identified by XRD.

The nanoscale morphology was then examined by electron microscopy. The HAADF‐STEM image in Figure 1d reveals numerous highly dispersed Sn‐containing nanoclusters distributed over the carbon‐black support. HRTEM further shows a lattice fringe with an interplanar spacing of approximately 0.28 nm, which is consistent with the Sn (101) plane (Figures 1e and S5) [55]. Particle‐size statistics for the catalyst series show a systematic increase in average particle size with increasing Sn precursor amount (Figure S5f). The low‐loading samples mainly contain smaller Sn‐containing domains, whereas Sn/SnO_x_‐16@CB exhibits a broader size distribution and more pronounced particle growth. In comparison, Sn/SnO_x_‐4@CB retains a relatively narrow particle‐size distribution centered at approximately 2–3 nm. Such a small nanocluster size is expected to provide a comparatively high proportion of exposed and low‐coordination Sn sites while limiting the particle aggregation observed at higher Sn loadings [56]. Elemental mapping further confirms the colocalized distribution of Sn and O within the carbon framework (Figure 1f), consistent with the coexistence of metallic and oxidized Sn‐containing domains at the nanoscale.

Sn K‐edge x‐ray absorption spectroscopy was further used to determine the average oxidation state and local coordination environment. The XANES edge position of Sn/SnO_x_‐4@CB lies between those of Sn foil and the oxidized Sn references and is closer to that of Sn foil (Figure 1g), confirming the coexistence of reduced and oxidized Sn species. The Fourier‐transformed EXAFS spectrum contains a Sn‐O contribution at approximately 1.5–1.6 Å and a Sn–Sn contribution at approximately 2.8–3.0 Å. Quantitative fitting gives a Sn‐O coordination number of 1.13 ± 0.26 with a bond length of 2.01 ± 0.01 Å and a Sn‐Sn coordination number of 3.29 ± 0.42 with a bond length of 3.01 ± 0.01 Å (Table S1). The low coordination numbers are consistent with the small particle size and the high proportion of undercoordinated sites. However, XPS and XAS cannot independently distinguish surface lattice oxygen from oxide species within the nanoparticles. Sn/SnO_x_‐4@CB is therefore conservatively described as nanoscale mixed Sn/SnO_x_. The simultaneous presence of Sn‐O and Sn‐Sn coordination is consistent with the mixed metallic/oxidized character indicated by XRD and XPS. Moreover, the relatively low fitted coordination numbers, compared with fully coordinated bulk reference structures, support the presence of small nanoclusters containing a high proportion of undercoordinated Sn sites. The wavelet‐transform EXAFS maps show both Sn‐O‐ and Sn‐Sn‐associated scattering regions for Sn/SnO_x_‐4@CB, whereas Sn foil is dominated by Sn‐Sn scattering and the oxide references exhibit pronounced Sn‐O scattering (Figure 1g). These results further support the coexistence of Sn‐Sn and Sn‐O local coordination within the Sn/SnO_x_‐4@CB sample. Electron paramagnetic resonance (EPR) spectroscopy was further employed to examine the electronic defects in the SnO_x_ component (Figure S6). The fresh Sn/SnO_x_@CB catalyst exhibited a distinct signal centered at g = 2.003, which is characteristic of unpaired electrons associated with oxygen‐vacancy‐related centers in metal oxides. The observation of this signal provides direct evidence for paramagnetic defect centers in the as‐prepared catalyst. After CO_2_ electrolysis, the signal remained detectable with a different intensity, suggesting that these centers were retained and that additional defect‐related states may have formed during cathodic surface reduction and reconstruction. This interpretation is consistent with the shift of the post‐electrolysis Sn 3d XPS spectrum toward lower binding energy.

These structural results define Sn/SnO_x_‐4@CB as defect‐rich Sn/SnO_x_ nanoclusters with nanoscale mixed Sn/SnO_x_ domains containing metallic and oxidized Sn species and a high fraction of undercoordinated sites. The metallic domain is expected to support electron transport, whereas the oxidized and undercoordinated surface sites can provide polar binding environments for oxygenated CO_2_‐reduction intermediates. This combination provides the structural basis for selective formic acid formation through the ^*^OCHO pathway.

### 3.2 Structure‐Sensitive Acidic CO_2_ Reduction Over Sn/SnO_x_‐4@CB

**FIGURE 2:**
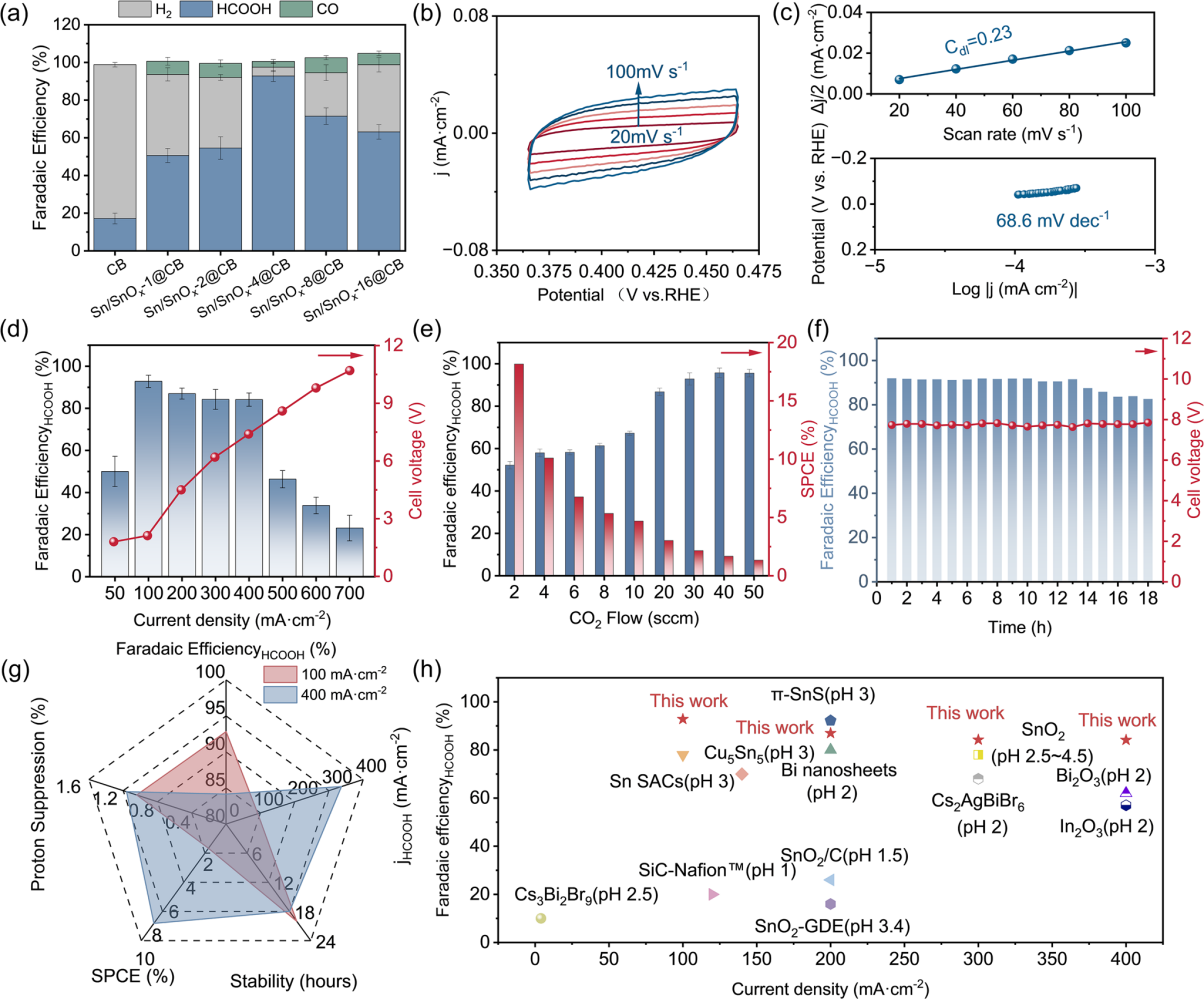
Electrocatalytic CO_2_ reduction performance of Sn/SnO_x_‐x@CB catalysts in acidic electrolyte. (a) Product Faradaic efficiencies of CB and Sn/SnO_x_‐x@CB catalysts at 100 mA cm^−2^. (b) Double‐layer capacitance values derived from cyclic voltammetry in the non‐Faradaic region for Sn/SnO_x_‐4@CB. (c) Tafel plots of Sn/SnO_x_‐4@CB catalysts. Figure 2a–c were measured in a membrane‐separated three‐electrode gas‐diffusion flow cell in CO_2_‐saturated 0.5 m K_2_SO_4_ (pH = 2, H_2_SO_4_). Electrolysis: 100 mA cm^−2^, CO_2_ flow 30 sccm, 25 mL electrolyte. Electrodes: catalyst‐coated gas‐diffusion (working), saturated KCl Ag/AgCl (reference), Pt plate (counter). (d) Faradaic efficiency for formic acid over Sn/SnO_x_‐4@CB as a function of current density. (e) Effect of CO_2_ flow rate on formic acid Faradaic efficiency and single‐pass carbon efficiency. (f) Chronopotentiometric stability test of Sn/SnO_x_‐4@CB in acidic K_2_SO_4_ electrolyte at 400 mA cm^−2^. (g) Comprehensive performance radar chart for formic acid electrosynthesis under different current densities. (h) Comparison of HCOOH Faradaic efficiency and current density for representative CO_2_ electrolysis systems operated with acidic catholytes. The comparison is restricted to studies in which the reported catholyte pH favors protonated HCOOH over HCOO^−^; neutral and alkaline formate‐producing systems are not included. Catholyte pH is indicated to account for differences in product speciation and HER competition.

The product distribution changed systematically with the Sn loading and nanocluster structure (Figure 2a). Bare CB mainly produced H_2_, with approximately 82% H_2_ and 17% HCOOH, whereas CO was negligible. After introducing Sn/SnO_x_, HCOOH became the dominant product, although a small amount of CO was detected. The HCOOH Faradaic efficiency increased from 50%–54% for Sn/SnO_x_‐1@CB and Sn/SnO_x_‐2@CB to 92% for Sn/SnO_x_‐4@CB, with corresponding CO efficiencies of approximately 7%, 8%, and 3%, respectively. Further increasing the loading decreased the HCOOH efficiency to approximately 71% and 63% for Sn/SnO_x_‐8@CB and Sn/SnO_x_‐16@CB, while H_2_ and CO formation increased. This volcano‐type dependence indicates that the catalytic performance is controlled by the balance among Sn/SnO_x_ interfacial sites, particle dispersion, and electronic conductivity rather than by the total Sn loading alone [57].

Electrochemical measurements further show that the catalytic performance does not scale directly with the relative capacitive response or apparent electrochemically accessible interface. Sn/SnO_x_‐4@CB exhibited a C_dl_ of 0.230 mF cm^−2^, higher than those of the lower‐loading samples but lower than those of Sn/SnO_x_‐8@CB and Sn/SnO_x_‐16@CB (Figures 2b and S7). Nevertheless, it delivered the highest HCOOH Faradaic efficiency among the catalyst series. Combined with the particle‐size analysis, these results suggest that the intermediate Sn loading provides a suitable balance between site accessibility and nanocluster dispersion. Further increasing the Sn loading enlarges the accessible interfacial area but also promotes particle growth, which may reduce the proportion of favorable low‐coordination and Sn/SnO_x_ interfacial sites.

Sn/SnO_x_‐4@CB also exhibited the lowest Tafel slope of 68.6 mV dec^−1^, compared with 82.0–136.76 mV dec^−1^ for the other samples (Figures 2c and S8), indicating more favorable apparent cathodic kinetics. Because the measured current may contain contributions from both CO_2_ reduction and HER, the Tafel slopes are used only for comparison rather than to assign a specific rate‐determining step. Consistently, Sn/SnO_x_‐4@CB exhibited the highest cathodic current density in the LSV measurements (Figure S9), indicating enhanced overall electrocatalytic activity. These results indicate that the superior performance of Sn/SnO_x_‐4@CB arises from an optimized active‐site structure and reaction kinetics rather than from surface area alone.

Electrochemical impedance spectroscopy was used to compare the interfacial impedance responses of the Sn‐loaded catalysts. The Nyquist plots were fitted using an *R_S_*‐(*R_ct_* ∥*CPE_dl_*) equivalent circuit, with the fitted curves and parameters provided in Figure S10 and Table S2, respectively. The fitted *R_ct_* values of Sn/SnO_x_‐1@CB, Sn/SnO_x_‐2@CB, Sn/SnO_x_‐4@CB, Sn/SnO_x_‐8@CB, and Sn/SnO_x_‐16@CB were 534.7, 132.4, 162.6, 193.0, and 332.7 Ω, respectively. Sn/SnO_x_‐2@CB therefore exhibited the lowest charge‐transfer resistance, whereas Sn/SnO_x_‐4@CB showed the second‐lowest value. Notably, the lower *R_ct_* of Sn/SnO_x_‐2@CB did not result in the highest formic acid Faradaic efficiency, demonstrating that the overall interfacial charge‐transfer resistance alone does not determine product selectivity. The superior formic acid production over Sn/SnO_x_‐4@CB is instead consistent with a balance among relatively favorable charge‐transfer behavior, nanocluster dispersion, accessible Sn/SnO_x_ sites, and selective stabilization of the *OCHO intermediate.

The electrocatalytic performance of Sn/SnO_x_‐4@CB was further evaluated in an acidic flow‐cell electrolyzer under industrially relevant current densities. As shown in Figure 2d, the Faradaic efficiency for HCOOH was 92.8% at 100 mA cm^−2^ and remained above 85% up to 400 mA cm^−2^. The corresponding cell voltage increased positively with increasing current density, reflecting the higher energy input required to drive CO_2_ electroreduction at elevated reaction rates. In Table S3, the HCOOH partial current density increased with the applied current density and reached 336.64 ± 12.60 mA cm^−2^ at 400 mA cm^−2^. The corresponding area‐normalized HCOOH production rate was 0.2890 ± 0.0108 g h^−1^ cm^−2^. At this current density, the HCOOH Faradaic efficiency was 84.16% ± 3.15%, demonstrating reproducible HCOOH production under high‐rate operating conditions. Above 400 mA cm^−2^, the declining HCOOH FE led to a decrease in the HCOOH production rate because of intensified competing reactions [58]. The lowest electrolyzer‐level specific electricity consumption was 2.66 ± 0.08 kWh kg^−1^ HCOOH at 100 mA cm^−2^. At 400 mA cm^−2^, the specific electricity consumption increased to 10.24 ± 0.38 kWh kg^−1^ HCOOH because of the higher cell voltage. Thus, operation at 100 mA cm^−2^ favored electrical energy efficiency, whereas 400 mA cm^−2^ provided the highest areal HCOOH productivity.

Electrolyte and flow‐rate effects confirm that local mass transport and interfacial ion structure are central to the observed selectivity. The formic acid Faradaic efficiency increased from 10% in dilute K_2_SO_4_ to nearly 98% in saturated K_2_SO_4_ (Figure S11b). The effect was especially pronounced above 0.1 m K_2_SO_4_, suggesting that cation concentration and interfacial hydration structure are involved in suppressing HER. The pH dependence showed an optimum at pH 2: selectivity was low at pH 1, increased to 92%–93% at pH 2, and then slightly decreased at higher pH (Figure S11a). This trend indicates that formic acid formation in acid requires a balance between suppressing excessive proton reduction and maintaining proton‐coupled CO_2_ reduction. CO_2_ flow rate showed a related effect. Increasing the flow rate from 2 to 50 mL min^−1^ raised the formic acid Faradaic efficiency from 52% to 96%, while the single‐pass carbon efficiency (SPCE) decreased (Figures 2e and S11c). The inverse relation between selectivity and single‐pass conversion is consistent with relief of local CO_2_ starvation at higher gas flow. For the 1 cm^2^ cathode used here, 2 sccm represents a transport‐sensitive condition with higher single‐pass CO_2_ utilization but stronger local CO_2_ depletion, whereas 30 sccm provides a CO_2_‐rich condition for evaluating catalyst performance with reduced external mass‐transfer limitation. As demonstrated by the optimization of 3 sccm in a 1 cm^2^ acidic PEM electrolyser and 300 sccm after scale‐up to 25 cm^2^, the practical CO_2_ feed must be adjusted according to electrode area, reaction rate, and reactor architecture rather than transferred as an absolute flow rate [7].

An initial stability test was then conducted at 400 mA cm^−2^ to assess the operational stability of the acidic CO_2_‐to‐HCOOH system. As displayed in Figure 2f, Sn/SnO_x_‐4@CB delivered a stable Faradaic efficiency of HCOOH of approximately 85% during 18 h of continuous operation. The cathodic potentials corresponding to the applied current densities of Sn/SnO_x_‐4@CB are provided in Table S4. The cathodic potential shifted from −1.256 V vs. RHE at 50 mA cm^−2^ to −2.667 V vs. RHE at 400 mA cm^−2^, and further to −3.345 V vs. RHE at 700 mA cm^−2^, reflecting increasing cathodic polarization at higher reaction rates. Extended electrolysis at 400 mA cm^−2^ maintained an HCOOH Faradaic efficiency above 85% for 18 h, with the cell voltage remaining at approximately 7.4 V without a progressive increase (Figures 2f and S12). In contrast, tests in KOH and KHCO_3_ showed lower selectivity and a gradual decline over the same period (Figure S13), highlighting that the acidic K_2_SO_4_ system is not merely tolerated but forms part of the optimized reaction environment. This demonstrates an initial stability of high‐current operation over the tested period, although longer tests are required to establish industrial durability. As shown in Figure 2g, the results reveal a trade‐off among selectivity, productivity, energy consumption, and single‐pass carbon efficiency. Operation at 100 mA cm^−2^ favors selectivity and energy efficiency, whereas 400 mA cm^−2^ provides the highest areal productivity. To unequivocally verify that the detected carbon products originated solely from the electroreduction of the supplied CO_2_ rather than the degradation of the carbon support or adventitious carbon sources, rigorous control experiments were conducted. When electrolysis was performed at a high current density of 400 mA cm^−2^ in an Ar‐saturated electrolyte, ion chromatography (IC) analysis showed no formate peak. In contrast, a massive formate signal was detected under a CO_2_ atmosphere (Figure S14). Similarly, gas chromatography (GC) confirmed that only H_2_ was produced under Ar, with no trace of CO or other carbonaceous gases (Figure S15). These results confirm that the carbon species in the products are exclusively derived from the continuously supplied CO_2_ reactant.

The negative shift of the Sn 3d XPS peaks (Figure S16) and the enhanced oxygen‐vacancy‐related EPR signal (Figure S6) indicate partial reduction and defect enrichment of the SnO_x_ surface during electrolysis. Despite this surface reconstruction, high HCOOH selectivity was maintained. In addition, the Janus substrate preserved its asymmetric wettability after electrolysis, suggesting that severe electrode flooding did not occur. Post‐electrolysis XRD showed that the principal Sn and SnO_2_ phases were retained without detectable new crystalline Sn‐containing phases (Figure S17), while the prominent PTFE peak originated from the gas‐diffusion substrate. The Sn content in the electrolyte increased from 0.0071 to 0.1083 ppm after electrolysis (Table S5), indicating detectable Sn dissolution or detachment. Thus, gradual Sn loss may contribute to long‐term deactivation. Other possible degradation pathways include nanoparticle coarsening, catalyst‐layer detachment, and progressive electrolyte intrusion, which require longer‐term testing and further post‐mortem characterization.

To ensure a chemically consistent comparison, Figure 2h includes reported CO_2_ electrolysis systems operated with acidic catholytes, with preference given to studies conducted below the p*K*_a_ of formic acid (p*K*_a_ = 3.75) and reporting HCOOH as the predominant liquid product [2, 8, 59, 60, 61, 62, 63, 64, 65, 66]. At the catholyte pH used here (pH 2), the Henderson‐Hasselbalch relationship indicates that approximately 98.3% of the HCOOH/HCOO^−^ product is present as protonated HCOOH. The present system combines an HCOOH Faradaic efficiency above 85% with a partial current density of 336.64 mA cm^−2^, demonstrating its advantage for high‐rate acidic operation. Future scale‐up must therefore reduce cell resistance and electrode spacing, improve the anodic reaction and membrane‐electrode architecture, and balance HCOOH selectivity against single‐pass carbon efficiency and product concentration.

### 3.3 Janus Wettability Control Stabilizes the Triple‐Phase Boundary

**FIGURE 3:**
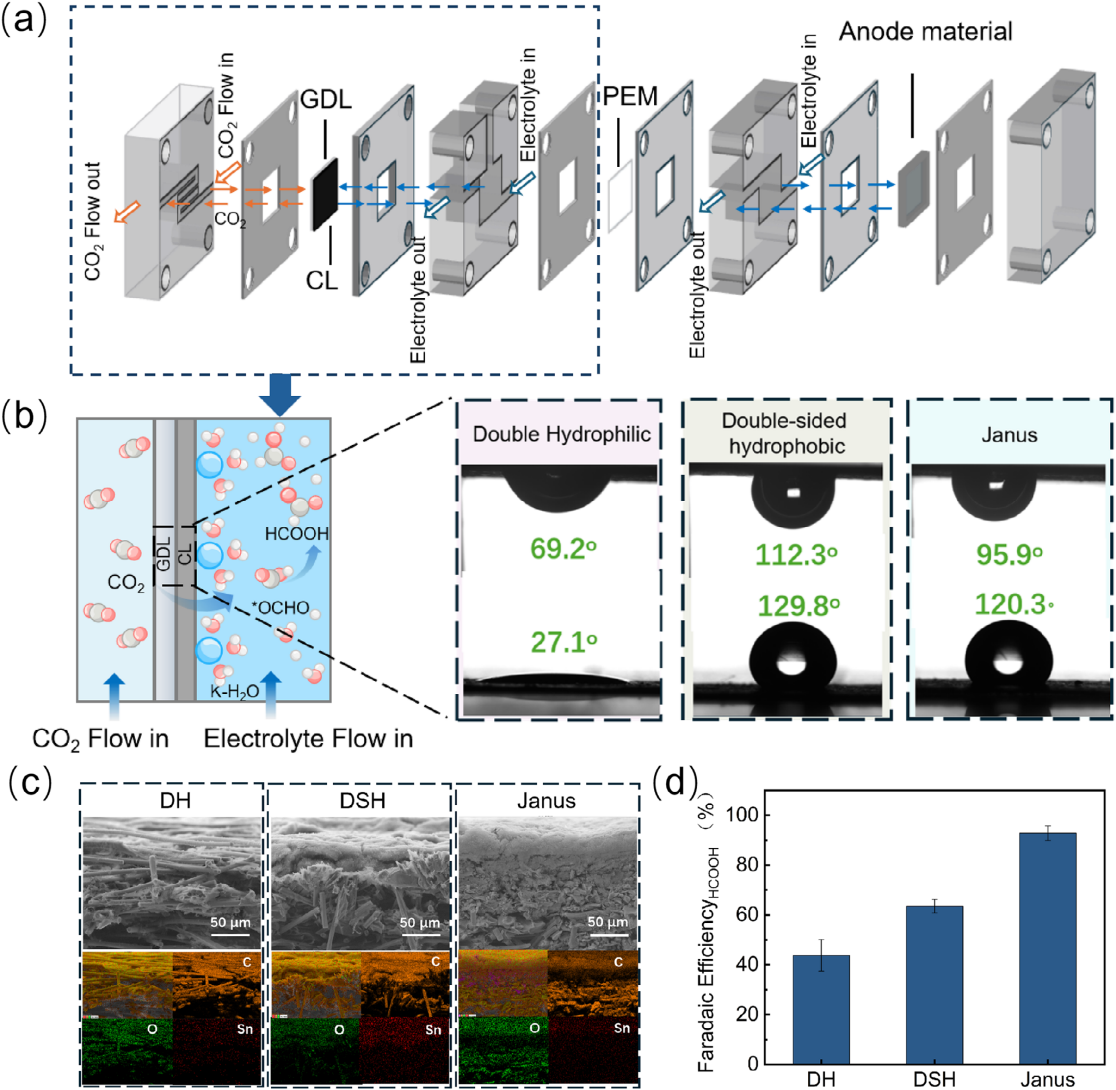
Regulation of the gas‐liquid‐solid interface by substrate wettability in acidic flow‐cell CO_2_ electroreduction. (a) Schematic illustration of the acidic flow‐cell electrolyzer and the cathodic gas‐diffusion electrode. (b) Underwater gas‐bubble contact angles measured on the electrolyte‐facing surfaces (upper row) and static water‐droplet contact angles measured in air on the gas‐facing surfaces (lower row) of the double‐hydrophilic substrate (DH), double‐sided hydrophobic substrate (DSH), and Janus asymmetric‐wettability substrate (Janus). The DSH substrate was prepared by depositing 0.75 mg cm^−2^ PTFE on each side, whereas the Janus substrate was prepared by depositing 0.75 mg cm^−2^ PTFE only on the gas‐facing side. (c) Cross‐sectional SEM images and elemental mapping of the electrodes prepared on the different substrates. (d) Formic acid Faradaic efficiencies of Sn/SnO_x_‐4@CB electrodes constructed on DH, DSH, and Janus substrates at 100 mA cm^−2^.

Although Sn/SnO_x_‐4@CB provides favorable intrinsic catalytic sites for CO_2_‐to‐formic acid conversion, its apparent performance in a flow cell is also governed by the stability of the gas‐liquid‐solid triple‐phase interface [67, 68]. As illustrated in Figure 3a, gaseous CO_2_ is supplied from the gas‐diffusion side, while the acidic electrolyte contacts the catalyst layer from the liquid side. The lower inset magnifies the gas diffusion electrode (GDE) interface, where gas transport, electrolyte penetration, and catalyst exposure jointly determine the stability of the gas‐liquid‐solid triple‐phase boundary. The reactor schematic has been revised to identify the gas chamber, catalyst‐coated gas‐diffusion cathode, catholyte chamber, proton‐exchange membrane, anolyte chamber, Pt anode, Ag/AgCl reference electrode, gas inlet and outlet, electrolyte reservoirs, circulation pumps, and product‐sampling positions in Figure S18.

An effective electrode should therefore allow continuous CO_2_ delivery through the porous gas pathway, maintain sufficient electrolyte contact for ionic conduction, and prevent excessive liquid penetration that would flood the gas‐diffusion layer. To clarify how electrode wettability regulates this interfacial balance, three types of substrates were compared: a double‐hydrophilic substrate (DH), a double‐sided hydrophobic substrate (DSH), and a Janus asymmetric‐wettability substrate. Contact‐angle measurements confirmed their distinct wetting behaviors (Figure 3b). The DH substrate exhibited contact angles of 69.2° on the gas side and 27.1° on the liquid side, indicating strong electrolyte affinity on both sides and, consequently, a high tendency toward electrolyte flooding. In contrast, the DSH substrate showed contact angles of 112.3° and 129.8°, suggesting that the hydrophobic surface can effectively resist electrolyte intrusion but may also restrict electrolyte access to catalytic sites. The Janus substrate displayed contact angles of 95.9° and 120.3° on the two sides, forming an asymmetric wetting structure. This configuration is expected to moderate electrolyte penetration from the liquid side while preserving open gas‐transport channels from the gas side, thereby stabilizing the triple‐phase boundary under flow‐cell operation. Post‐electrolysis wettability measurements were conducted to evaluate the retention of the asymmetric wetting characteristics of the Janus electrode. After operation at 400 mA cm^−2^ for 18 h, the hydrophobic gas‐facing side exhibited a water contact angle of 121.3° in air and an underwater captive‐bubble angle of 79.4°, whereas the relatively wettable electrolyte‐facing side showed corresponding values of 94.1° and 34.6°, respectively (Figure S19). The captive‐bubble angles were defined through the aqueous phase outside the bubble. Both measurement configurations therefore yielded the same wettability ordering between the two sides. Moreover, compared with the pre‐electrolysis water contact angles of 120.3° and 95.9°, respectively, only minor changes of +1.0° and −1.8° were observed after operation. These ex situ results support the retention of the macroscopic wetting asymmetry of the Janus architecture, although they do not directly resolve the dynamic liquid distribution inside the operating porous electrode.

Cross‐sectional SEM images and elemental mapping further support this interpretation. For the DH substrate, Sn‐ and O‐containing regions penetrated deeply into the porous framework, consistent with electrolyte‐driven catalyst‐layer migration and partial flooding of the electrode (Figure 3c). By comparison, the catalyst layer on the Janus substrate remained more confined near the designed reaction interface, and the underlying porous structure was better preserved. Surface and cross‐sectional SEM images of the different electrodes also confirmed that the Janus electrode retained a more uniform and less collapsed porous morphology than the DH electrode after electrode preparation and operation (Figure S20).

The electrochemical results are consistent with the structural observations. At 100 mA cm^−2^, the DH electrode showed limited formic acid Faradaic efficiency because severe electrolyte penetration blocked CO_2_ transport and promoted proton reduction (Figure 3d). The DSH electrode improved the selectivity by suppressing flooding, but the overly hydrophobic interface could limit electrolyte wetting, ionic conduction, and active‐site utilization. In contrast, the Janus electrode delivered the highest formic acid Faradaic efficiency, indicating that neither complete hydrophilicity nor excessive hydrophobicity is optimal. Instead, the asymmetric‐wettability design provides a more balanced gas‐liquid‐solid interface, where CO_2_ supply, electrolyte access, and catalyst utilization are simultaneously maintained.

The Janus substrate provides a better balance between electrolyte access and CO_2_ transport than the uniformly wettable or hydrophobic electrodes. Microscopy and post‐electrolysis contact‐angle measurements further support reduced electrolyte intrusion and retention of the wetting asymmetry. It should be regarded not merely as a passive support, but as an integral component of the catalytic system that stabilizes the triple‐phase boundary and enables selective formic acid production in acidic flow‐cell electrolysis.

### 3.4 K^+^‐Specific Interfacial Regulation Promotes Formic Acid Formation

**FIGURE 4:**
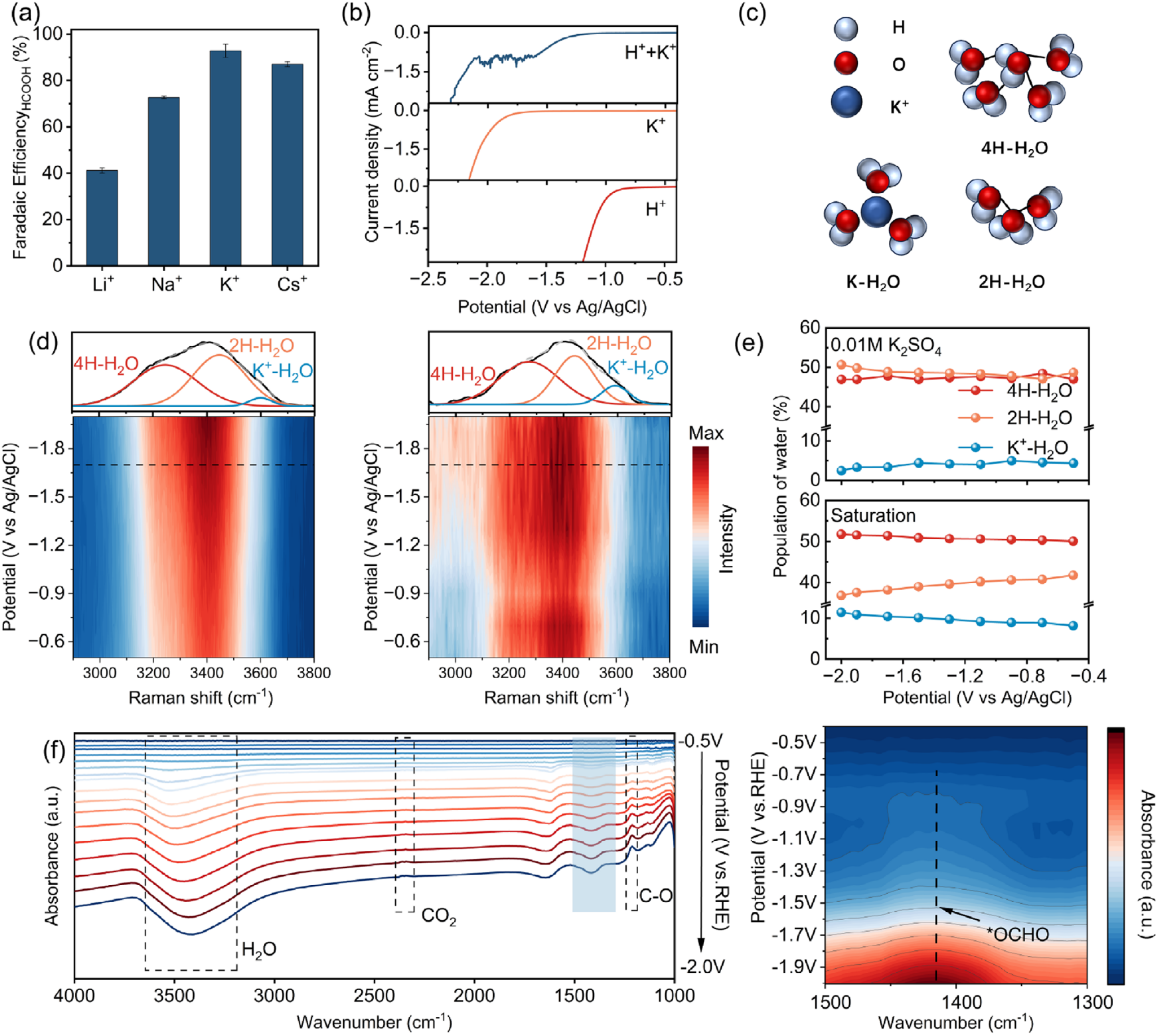
Concentration‐dependent interfacial‐water response and cation‐dependent CO_2_‐reduction selectivity in acidic electrolytes. (a) Cation‐dependent product Faradaic efficiencies over Sn/SnO_x_‐4@CB in acidic sulfate electrolytes containing Li^+^, Na^+^, K^+^, or Cs^+^. (b) LSV curves collected under different electrolyte environments. (c) Representative operando Raman spectra in the O‐H stretching region collected during CO_2_ electroreduction. (d) Deconvolution of the O‐H stretching band into water species assigned to strongly hydrogen‐bonded 4H‐H_2_O, weakly or partially hydrogen‐bonded 2H‐H_2_O, and K^+^‐coordinated water. (e) Potential‐dependent evolution of the fitted O‐H stretching components in dilute (0.01 m) and saturated K_2_SO_4_ electrolytes. (f) In situ ATR‐FTIR spectra collected during CO_2_ electroreduction over Sn/SnO_x_‐4@CB.

The strong dependence on K_2_SO_4_ concentration suggests that K^+^ is involved in regulating the local reaction environment rather than serving only as a supporting electrolyte ion. To examine this effect, alkali‐metal cations were compared under otherwise similar acidic conditions. The formic acid Faradaic efficiency followed the order K^+^ > Cs^+^ > Na^+^ > Li^+^ (Figure 4a). Li^+^ gave only 20% selectivity, Na^+^ improved the selectivity to 35%–40%, K^+^ produced nearly quantitative formic acid selectivity, and Cs^+^ decreased the value to 75%–80%. The nonmonotonic trend cannot be explained by ionic strength or unsolvated ionic radius alone. Recent Raman‐based studies of acidic CO_2_RR have shown that alkali‐metal size can simultaneously affect intermediate binding and the interfacial water network [69, 70]. The observed K^+^ optimum therefore likely reflects a balance among cation hydration, interfacial accumulation, water‐network reorganization, and adsorbate stabilization, rather than a monotonic dependence on Lewis acidity or ionic radius.

LSV measurements further showed that the cathodic response changed substantially when K^+^ was introduced into the acidic electrolyte (Figure 4b). In a proton‐rich electrolyte without effective cation regulation, the current was dominated by HER. In the presence of K^+^, the current response shifted in a way consistent with greater CO_2_‐reduction contribution and suppressed proton reduction. This observation is consistent with the product distribution but does not by itself identify the molecular origin of the effect.

Operando Raman spectroscopy was used to examine whether the interfacial O‐H stretching response depends on the concentration of K_2_SO_4._ Dilute and saturated K_2_SO_4_ electrolytes were selected to represent two limiting concentration regimes. Gaussian deconvolution of the broad O‐H stretching envelope yielded three representative components associated with strongly hydrogen‐bonded four‐coordinated water (4H‐H_2_O), less extensively hydrogen‐bonded two‐coordinated water (2H‐H_2_O), and water influenced by K^+^ coordination (K‐H_2_O), respectively (Figures 4c,d and S21). Relative to the dilute electrolyte, the saturated electrolyte exhibited a more pronounced potential‐dependent redistribution of these fitted components, while the fraction assigned to strongly hydrogen‐bonded water remained approximately 50%–52% (Figure 4e). These observations demonstrate that the interfacial‐water response is sensitive to K_2_SO_4_ concentration and applied potential. The involvement of K^+^associated solvation and broader electrolyte environmental factors, including ionic strength, water activity, and ion pairing, regulates the interfacial hydrogen‐bond network, which may consequently modulate proton transfer and the stabilization of polar CO_2_ reduction intermediates [69]. The Faradaic efficiency for formic acid increases markedly with increasing K_2_SO_4_ concentration and reaches 92% in 0.5 m K_2_SO_4_, which is close to the 95% obtained in saturated K_2_SO_4_. Thus, the standard 0.5 m K_2_SO_4_ electrolyte already lies in the high‐selectivity concentration regime. Taken together, the Raman and electrochemical results support an association among electrolyte concentration, interfacial‐water organization, and suppression of HER.

In situ ATR‐FTIR spectroscopy further identifies the reaction intermediate associated with formic acid formation under the K_2_SO_4_ operating environment. As the cathodic potential became more negative, the absorption band assigned to *OCHO developed progressively, together with signals associated with oxygenated carbon species (Figure 4f). The potential‐dependent accumulation of OCHO, a key intermediate for formic acid production on Sn‐based catalysts, confirms that CO_2_ reduction over Sn/SnO_x_‐4@CB proceeds through the desired O‐bound intermediate pathway [71]. Together, these electrochemical activity and spectroscopic results show that the high formic acid selectivity in the K^+^‐containing electrolyte coincides with interfacial‐water reorganization and potential‐dependent OCHO accumulation, providing molecular‐level support for the proposed K^+^‐regulated reaction microenvironment.

### 3.5 Theoretical Analysis of Alkali‐Metal Effects on the Simplified Model

**FIGURE 5:**
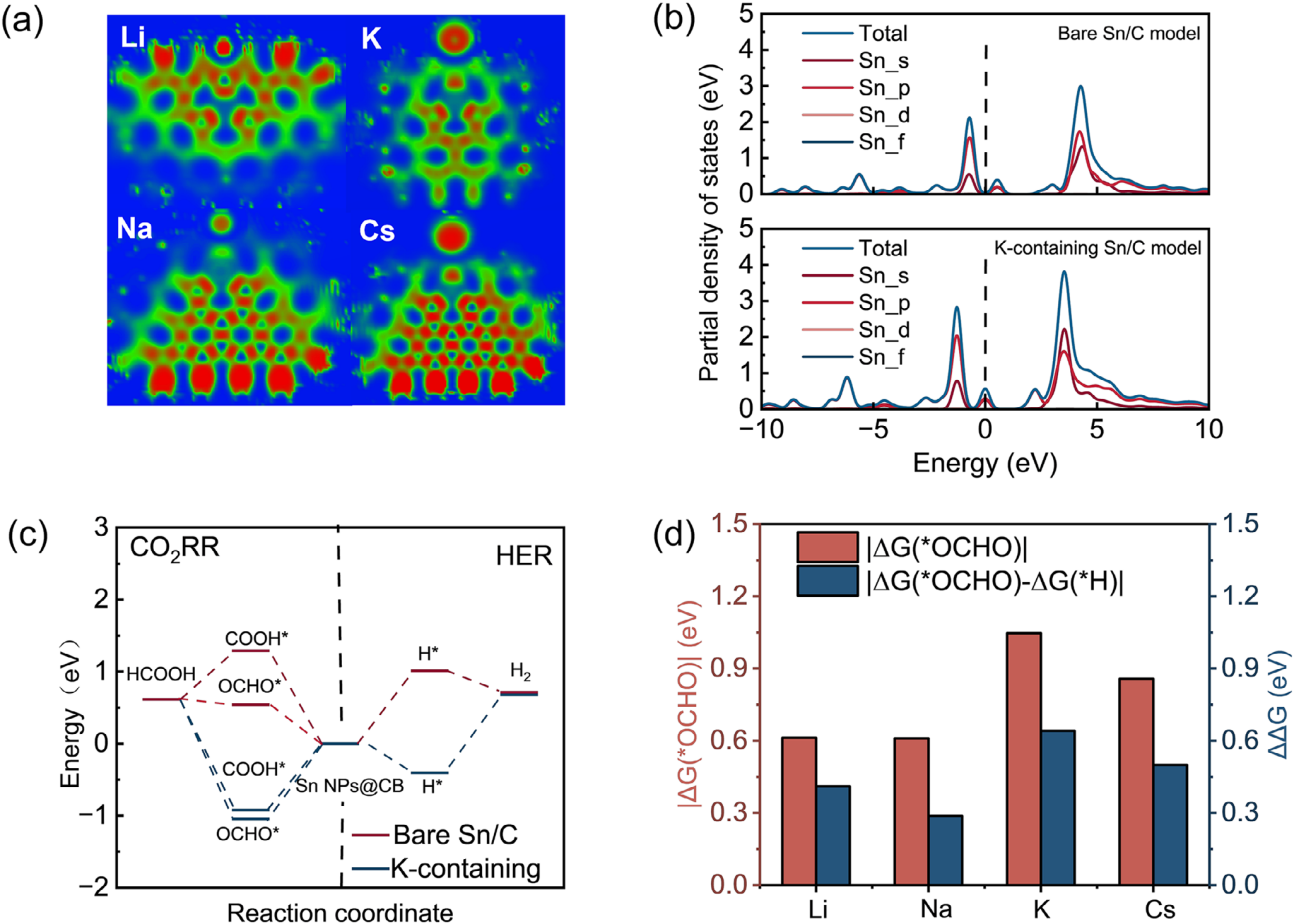
Alkali‐metal‐dependent electronic structures and adsorption energetics on a simplified carbon‐supported Sn model. (a) Electron localization function maps of the Li‐, Na‐, K‐, and Cs‐containing Sn/C models. All maps are presented using the same color scale and section definition. (b) Projected density of states of the bare and K‐containing Sn/C models, with the Fermi level set to zero. (c) Calculated free‐energy profiles of the *OCHO and *COOH routes and the competing *H route on the bare and K‐containing Sn/C models. (d) Calculated ‐G(*OCHO) and G(*H)‐G(*OCHO) values for the Li‐, Na‐, K‐, and Cs‐containing models. A larger positive G(*H)‐G(*OCHO) value indicates a greater thermodynamic preference for *OCHO over *H.

DFT calculations were performed to compare the influence of nearby alkali‐metal species on the adsorption energetics of *COOH, *OCHO, and *H using a common carbon‐supported Sn model. The catalyst framework of the computational model contains Sn and C; it therefore represents a simplified Sn/C adsorption motif. Li, Na, K, and Cs were introduced separately near the Sn adsorption region while retaining the same Sn/C framework. Accordingly, the calculations are used to evaluate alkali‐metal‐dependent trends. The electron localization function maps exhibit distinct spatial localization patterns for the Li‐, Na‐, K‐, and Cs‐containing Sn/C models (Figure 5a and Table S6). The differences are mainly observed around the Sn‐containing adsorption region and the nearby alkali‐metal species, indicating that the local electronic environment of the Sn motif is related to the identity and optimized configuration of the introduced alkali metal. The K‐containing model displays a localization pattern different from those of the Li‐, Na‐, and Cs‐containing models. The projected density of states further indicates that the electronic structure of the Sn motif is perturbed by the introduction of K (Figures 5b and S22). Compared with the bare Sn/C model, the K‐containing model exhibits changes in the distribution and intensity of Sn‐derived states on both sides of the Fermi level. These differences are consistent with a modified local electronic environment around the Sn adsorption motif. However, the PDOS is treated as a qualitative electronic‐structure descriptor; the relative thermodynamic preference among the adsorbed intermediates is evaluated from the calculated free energies.

On the bare Sn/C model, the calculated free energies of *OCHO and *COOH are 0.540 and 1.290 eV, respectively (Figures 5c and S23). Thus, *OCHO is lower in free energy than *COOH by 0.750 eV under the adopted computational reference conditions. This result indicates a thermodynamic preference for the *OCHO configuration over the competing *COOH configuration on the simplified Sn/C motif. Introduction of the alkali‐metal species changes the calculated free energies of the adsorbed intermediates. Among the examined models, the K‐containing configuration gives the lowest absolute free energies for both *OCHO and *COOH, whereas the preference of *OCHO over *COOH is retained for all four alkali‐metal species. Also, the introduction of the alkali‐metal species lowers the calculated free energy of *H as well as that of *OCHO (Table S7). The relevant distinction is the relative free‐energy separation between *OCHO and *H. The values of G(*H)‐G(*OCHO) are 0.411, 0.288, 0.642, and 0.500 eV for the Li‐, Na‐, K‐, and Cs‐containing models, respectively (Figure 5d). The K‐containing configuration therefore exhibits the largest thermodynamic preference for *OCHO over *H among the four alkali‐metal‐containing models. For comparison, this separation is 0.470 eV on the bare Sn/C model. The introduction of K increases the separation by 0.172 eV relative to the bare model. K species provides the most favorable formic acid selectivity among the examined alkali‐metal cations.

The theoretical and spectroscopic results therefore address complementary aspects of the cation effect. The simplified DFT model indicates that the identity of the nearby alkali‐metal species changes the relative thermodynamics of *OCHO and *H adsorption on an Sn/C motif. The concentration‐dependent interfacial‐water reorganization is supported by operando Raman spectroscopy. Taken together, the results suggest that the experimentally observed K^+^ effect may involve both cation‐dependent adsorbate interactions and changes in the interfacial solvation environment [64, 72].

### 3.6 Prospective Life‐Cycle and Techno‐Economic Implications

A prospective cradle‐to‐gate screening assessment was conducted to examine the environmental and economic implications of the present acidic CO_2_‐to‐HCOOH over the Sn/SnO_x_‐4@CB catalyst accounting frameworks for electrochemical CO_2_ conversion systems (Figure S24). The base case was anchored to the experimentally demonstrated operation at 400 mA cm^−2^. The HCOOH partial current density of 336.64 mA cm^−2^ corresponds to an FE of 84.16%, while the cell voltage and areal HCOOH production rate were approximately 7.4 V and 0.289 g cm^−2^ h^−1^, respectively (Table S8).

The electrolyzer‐level electricity demand was 10.24 kWh kg^−1^ HCOOH. Including auxiliary operation and a screening separation energy of 5.0 kWh kg^−1^ gave a total electricity requirement of 16.26 kWh kg^−1^ HCOOH. The separation term was treated as a scenario parameter because the outlet HCOOH concentration and detailed recovery process were not determined experimentally (Table S9).

Under the grid‐electricity base case, the gross cradle‐to‐gate GWP was estimated to be 9.14 kg CO_2_e kg^−1^ HCOOH. Electrolysis electricity contributed 5.63 kg CO_2_e kg^−1^, while auxiliary operation and product separation contributed 0.56 and 2.75 kg CO_2_e kg^−1^, respectively. Together, these terms accounted for approximately 98% of the total GWP. The contribution from catalyst synthesis was only about 0.007 kg CO_2_e kg^−1^ HCOOH, even when catalyst use was conservatively allocated over the demonstrated 18 h operating period.

**FIGURE 6:**
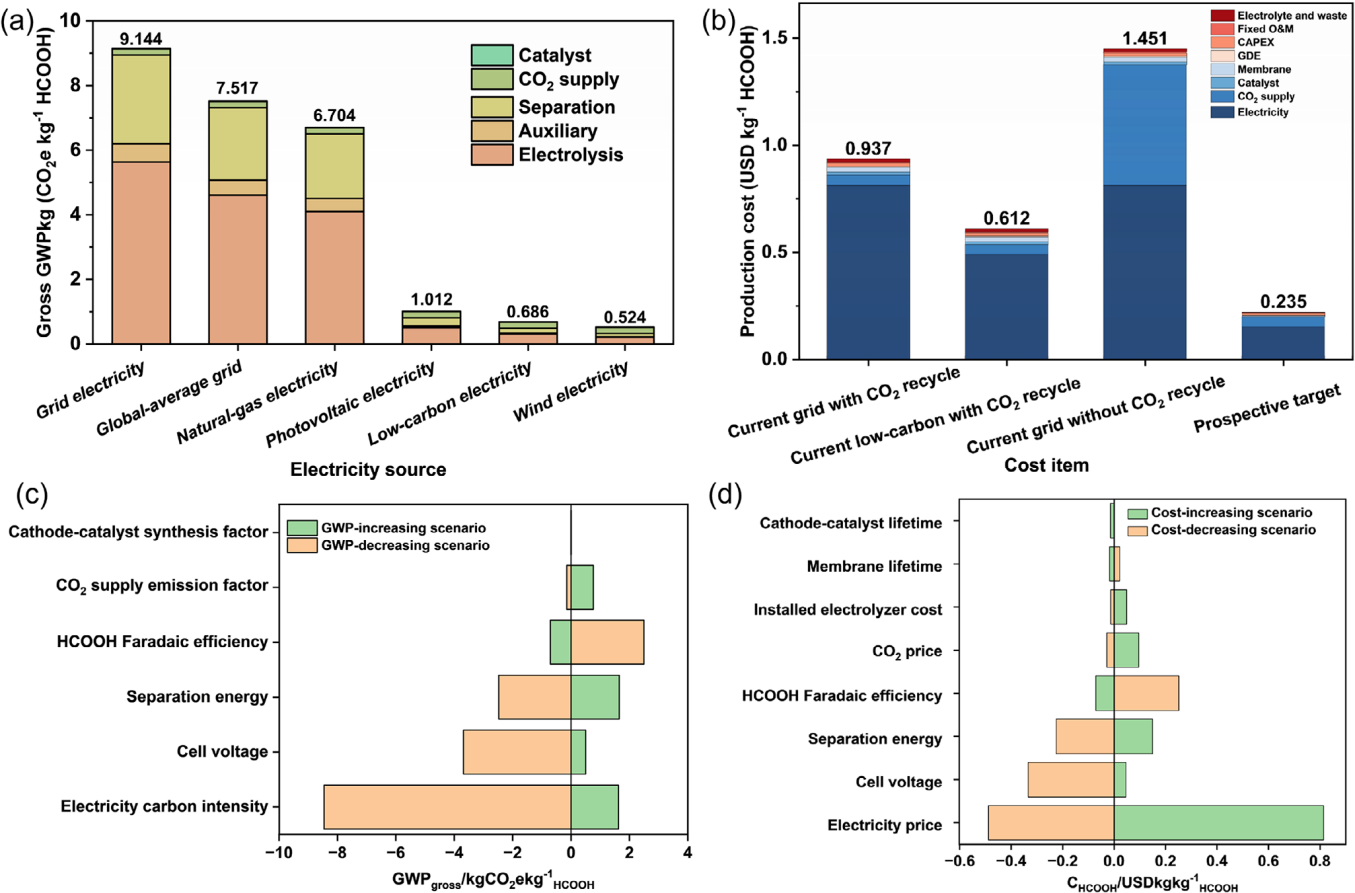
Prospective environmental and economic assessment of acidic CO_2_‐to‐HCOOH electrolysis. (a) GWP breakdown under different electricity sources. (b) Production‐cost breakdown for the current and prospective scenarios. Sensitivity of (c) GWP and (d) production cost to key operating and economic parameters. The prospective target is an engineering scenario rather than experimentally demonstrated performance.

The electricity source strongly affected the result. Replacing the grid‐electricity factor of 0.55 kg CO_2_e kWh^−1^ with a low‐carbon factor of 0.03 kg CO_2_e kWh^−1^ reduced the estimated gross GWP from 9.14 to 0.69 kg CO_2_e kg^−1^ HCOOH (Figure 6a). No permanent carbon‐sequestration credit was assigned to the CO_2_ contained in HCOOH because downstream use may release the carbon.

The screening‐level TEA gave an estimated production cost of approximately 0.94 USD kg^−1^ HCOOH for a nominal 10 000 t yr^−1^ plant using grid electricity at 0.05 USD kWh^−1^ and CO_2_ recycling. Electricity, including electrolysis, auxiliary operation, and product recovery, contributed approximately 0.81 USD kg^−1^ and represented about 87% of the total cost. The catalyst contribution was approximately 0.014 USD kg^−1^. Low‐carbon electricity at 0.03 USD kWh^−1^ reduced the estimated cost to approximately 0.61 USD kg^−1^ (Figure 6b and Table S10).

CO_2_ recycling was also important because the calculated single‐pass carbon efficiency under the 30 sccm base condition was only approximately 8.5%. Without recycling, the estimated production cost increased to approximately 1.45 USD kg^−1^. In a prospective target case combining 95% HCOOH FE, 500 mA cm^−2^, a cell voltage of 3.0 V, a separation energy of 1.0 kWh kg^−1^, low‐carbon electricity, and CO_2_ recycling, the projected cost decreased to approximately 0.24 USD kg^−1^. This target value is not an experimentally achieved industrial cost. It identifies the performance improvements required for practical development (Figure 6c,d and Table S10).

## 4 Conclusions

This work couples active‐site, solvation, and transport regulation for acidic HCOOH electrosynthesis. Defect‐rich Sn/SnO_x_ nanoclusters promote the *OCHO pathway, K^+^‐dependent interfacial regulation reduces *H competition, and the Janus electrode balances CO_2_ transport with electrolyte access. The system achieves 92.8% HCOOH Faradaic efficiency at 100 mA cm^−2^ and above 85% at 400 mA cm^−2^. At 400 mA cm^−2^, the HCOOH partial current density is 336.64 mA cm^−2^ and the production rate is 0.289 g h^−1^ cm^−2^, with an initial stability of 18 h. The main advance is the coordinated suppression of HER from the catalyst site to the gas‐diffusion electrode. Further work should focus on scaling up membrane‐electrode assemblies, reducing cell resistance, increasing product concentration and carbon utilization, and evaluating long‐term stability under practical operating conditions. This study provides a design strategy for selective HCOOH production under acidic, high‐current‐density conditions.

## Author Contributions

**Qiaoqi Guo**: writing – original draft, data curation, methodology, visualization. **Bowen Jiang**: methodology, data curation, visualization. **Le Huang**: methodology, visualization. **Kunyu Jiang**: data curation, investigation. **Rongrong Tang**: data curation, investigation. **Qinong Zhu**: data curation, investigation. **Huajun Feng**: resources, supervision. **Mengyang Fan**: writing – review and editing. **Yijing Xia**: funding acquisition, conceptualization, writing – review and editing.

## Conflicts of Interest

The authors declare no conflicts of interest.

## Supporting information




**Supporting File**: advs77692‐sup‐0001‐SuppMat.docx.

## Data Availability

The data that support the findings of this study are available from the corresponding author upon reasonable request.
