# Supplementary material for: High‐Current‐Density Acidic CO2 Electroreduction to Formic Acid Enabled by Multiscale Microenvironment Regulation Over Defect‐Rich Sn/SnOx
Source: Adv Sci (Weinh). 2026 Sep 8:e77692. Online ahead of print. doi: 10.1002/advs.77692 (PMC13554383; doi:10.1002/advs.77692)
Supplement: Supplementary file 1 [file ADVS-9999-e77692-s001.docx]

**High-Current-Density Acidic CO_2_ Electroreduction to Formic Acid Enabled by Multiscale Microenvironment Regulation over Defective Sn/SnO_x_**

Qiaoqi Guo^a,b^, Bowen Jiang^a^, Le Huang^c^, Kunyu Jiang^a^, Rongrong Tang^a^, Qinong Zhu^a^, Huajun Feng^b^, Mengyang Fan^d^, Yijing Xia^a*^

^a^ Zhejiang Key Laboratory of Solid Waste Pollution Control and Resource Utilization, School of Environmental Science and Engineering, Zhejiang Gongshang University, Hangzhou 310018, China

^b^ College of Environment and Resources, College of Carbon Neutral, Zhejiang A&F University, Hangzhou 311300, Zhejiang, China

^c^ Jinhua Academy of Zhejiang Chinese Medical University, Jinhua 321015, Zhejiang,

China

^d^ College of Carbon Neutrality Future Technology, Sichuan University, Chengdu 610065, China

*Corresponding author:

Yijing Xia, Email: xiayj@zjsu.edu.cn

**Figure S1** Morphology and elemental distribution of the Sn/SnO_x_-4@CB catalyst. (a-c) Representative SEM images of Sn/SnO_x_-4@CB recorded at different magnifications. The scale bars are 1 μm in (a), 500 nm in (b), and 100 nm in (c). (d-f) EDS elemental maps of a representative region of Sn/SnO_x_-4@CB, showing the spatial distributions of (d) C, (e) O, and (f) Sn. C, O, and Sn are displayed in orange, green, and red, respectively. The scale bars in (d-f) are 1 μm.

**Figure S2** XRD patterns of a series of Sn/SnO_x_-x@CB catalysts.

**Figure S3** XPS survey and Sn 3*d* spectra of CB and Sn/SnO_x_-x@CB catalysts. (a) XPS survey spectra confirming the presence of C, O, and Sn in Sn-loaded catalysts. (b) High-resolution Sn 3*d* spectra of Sn/SnO_x_-x@CB catalysts.

**Figure S4** High-resolution O 1*s* XPS spectra of Sn/SnO_x_-x@CB catalysts.

**Figure S5** HRTEM characterization and particle-size statistics of Sn/SnO_x_-x@CB catalysts. (a-e) Representative HRTEM images of (a) Sn/SnO_x_-1@CB, (b) Sn/SnO_x_-2@CB, (c) Sn/SnO_x_-4@CB, (d) Sn/SnO_x_-8@CB, and (e) Sn/SnO_x_-16@CB. The insets show inverse fast Fourier transform (IFFT) images obtained from the corresponding boxed regions. The observed lattice fringes are assigned to the Sn (101) plane, with interplanar spacings of 0.283, 0.282, 0.283, 0.282, and 0.282 nm for Sn/SnO_x_-1@CB, Sn/SnO_x_-2@CB, Sn/SnO_x_-4@CB, Sn/SnO_x_-8@CB, and Sn/SnO_x_-16@CB, respectively. The orange parallel lines and arrows in the insets indicate the measured lattice periodicity. The scale bars in (a-e) are 5 nm. (f) Particle-size distributions of the Sn-containing nanoclusters in the five catalyst samples; the red curves represent Gaussian fits to the experimental distributions.

**Figure S6** Electron paramagnetic resonance spectra of the optimized Sn/SnO_x_-4@CB catalyst before (Fresh) and after (Used) acidic CO_2_ electrolysis.

**Figure S7** Cyclic voltammograms collected in a non-Faradaic potential region at different scan rates and the corresponding linear relationships between the capacitive current-density difference and scan rate. Determination of the double-layer capacitances of the Sn/SnO_x_-x@CB catalyst series. C_dl_ was obtained from the slope of (j_a_ - j_c_)/2 plotted against scan rate. All measurements were performed under identical electrode-preparation and electrochemical conditions.

**Figure S8** Tafel analysis of the Sn/SnO_x_-x@CB catalyst series.

**Figure S9** Linear sweep voltammetry of CB and Sn/SnO_x_-x@CB catalysts.

**Figure S10** Electrochemical impedance spectroscopy of CB and Sn/SnO_x_-x@CB catalysts. Nyquist plots measured in CO_2_-saturated acidic electrolyte.

**Figure S11** Effects of electrolyte composition, pH, and CO_2_ flow rate on CO_2_ electroreduction over Sn/SnO_x_-4@CB. (a) pH-dependent Faradaic efficiencies for formic acid and H_2_. (b) Effect of K_2_SO_4_ concentration on product distribution. (c) Single-pass carbon efficiency as a function of CO_2_ flow rate.

**Figure S12** Chronopotentiometric stability test of Sn/SnO_x_-4@CB in acidic K_2_SO_4_ electrolyte at 100 mA cm^-2^

**Figure S13** Stability comparison in different electrolyte environments. Chronopotentiometric stability tests and product Faradaic efficiencies of Sn/SnO_x_-4@CB in acidic K_2_SO_4_, KHCO_3_, and KOH electrolytes.

**Figure S14** Ion chromatograms of the electrolyte solutions collected after electrolysis under Ar (blue line) and CO_2_ (red line) atmospheres. Sn/SnO_x_-4@CB were tested at a current density of 400 mA·cm^-2^ in 0.5 M K_2_SO_4_ electrolyte at pH 2.

**Figure S15** (a, b) FID and TCD gas chromatograms of Sn/SnO_x_-4@CB under a CO_2_ atmosphere; (c, d) FID and TCD gas chromatograms of Sn/SnO_x_-4@CB under an Ar atmosphere. Sn/SnO_x_-4@CB were tested at a current density of 400 mA·cm^-2^ in 0.5 M K_2_SO_4_ electrolyte at pH 2.

**Figure S16** Post-electrolysis XPS analysis of Sn/SnO_x_-4@CB. High-resolution Sn 3d XPS spectra of Sn/SnO_x_-4@CB before and after acidic CO_2_ electrolysis.

**Figure S17** Post-electrolysis XRD characterization of Sn/SnO_x_-4@CB electrode. XRD patterns of Sn/SnO_x_-4@CB before and after electrolysis.

**Figure S18** Schematic illustration of the flow cell setup for CO_2_ electroreduction. A catalyst-coated gas diffusion electrode (GDE) serves as the cathode, paired with a Pt anode and an Ag/AgCl reference electrode. A proton-exchange membrane separates the catholyte and anolyte compartments. CO_2_ is supplied to the gas chamber adjacent to the GDE. Catholyte and anolyte circulate continuously.

**Figure S19** Post-electrolysis wettability of the Janus electrode. Underwater captive-bubble contact angles of (a) the hydrophobic gas-facing side and (c) the relatively wettable electrolyte-facing side; static water contact angles measured in air on (b) the hydrophobic gas-facing side and (d) the relatively wettable electrolyte-facing side. The underwater captive-bubble contact angles were measured through the aqueous phase outside the bubble.

**Figure S20** Morphology of electrodes with different wettability configurations. Top-view and cross-sectional SEM images of double-hydrophilic (DH), double-sided hydrophobic (DSH), and Janus electrodes (Janus).

**Figure S21** Operando Raman analysis of interfacial water structures in K_2_SO_4_ electrolytes. (a) Potential-dependent Raman spectra and Gaussian fitting of the O-H stretching region in dilute K_2_SO_4_. (b) Corresponding spectra and fitted components in saturated K_2_SO_4_.

**Figure S22** Projected density of states of Bare Sn/C and Sn/C models containing alkali metals.

**Figure S23** Calculated free-energy profiles on the bare and alkali-metal-containing Sn/C models. (a) Bare Sn/C model; (b) Li-containing Sn/C model; (c) Na-containing Sn/C model; (d) K-containing Sn/C model; and (e) Cs-containing Sn/C model. The catalyst framework contains no lattice oxygen or explicit SnO_x_ phase. The values used to construct the profiles are summarized in Table S4.

**Figure S24** Cradle-to-gate system boundary of prospective environmental and economic assessment of acidic CO_2_-to-HCOOH electrolysis.

**Table S1 EXAFS fitting parameters at the Sn K‑edge for Sn‑foil reference and the sample.**

| Sample | Path | N | R（Å） | σ^2^（🞨10^-3^ Å^2^） | E_0_  （eV） | R-factor |
| --- | --- | --- | --- | --- | --- | --- |
| Sn foil | Sn-Sn | 4 | 2.99+/-0.006 | 10.61+/-0.71 | 5.71+/- 0.47 | 0.01 |
| sample | Sn-O | 1.13(0.26) | 2.01(0.01) | 8.81(4.07) | 5.17(0.79) | 0.017 |
|  | Sn-Sn | 3.29(0.42) | 3.01(0.01) | 11.86(1.61) |  |  |

**Table S2 EIS fitting parameters of Sn/SnO_x_-x@CB catalysts obtained using the** $\text{R}_{\text{S}}\text{−(}\text{R}_{\text{ct}}\text{∥}\text{CPE}_{\text{dl}}\text{)}$ **equivalent circuit**

| Sample | $\text{R}_{\text{s}}$(Ω) | $\text{R}_{\text{ct}}$(Ω) | Q(S s^n^) | n | $\chi^{\text{2}}$ |
| --- | --- | --- | --- | --- | --- |
| Sn/SnO_x_-1@CB | 2.399 ± 0.030 | 534.7 ± 14.6 | (1.5888 ± 0.0353) × 10^-3^ | 0.8018 ± 0.0051 | 0.023341 |
| Sn/SnO_x_-2@CB | 2.804 ± 0.041 | 132.4 ± 6.9 | (2.0707 ± 0.0972) × 10^-3^ | 0.7464 ± 0.0093 | 0.029715 |
| Sn/SnO_x_-4@CB | 2.314 ± 0.027 | 162.6 ± 4.4 | (1.1977 ± 0.0374) × 10^-3^ | 0.7464 ± 0.0056 | 0.010956 |
| Sn/SnO_x_-8@CB | 2.498 ± 0.028 | 193.0 ± 6.8 | (2.5431 ± 0.0688) × 10^-3^ | 0.7500 ± 0.0059 | 0.014341 |
| Sn/SnO_x_-16@CB | 2.493 ± 0.046 | 332.7 ± 21.4 | (5.1421 ± 0.1698) × 10^-3^ | 0.7604 ± 0.0093 | 0.076692 |

**Table S3 Electrochemical performance and energy consumption of CO_2_ electroreduction to formic acid under different total current densities**

| Total current density  (mA cm^-2^) | *j_HCOOH_*  (mA cm^-2^) | *FE_HCOOH_*  (%) | Cell voltage  (V) | HCOOH  Production rate  (mmol h^-1^ cm^-2^) | HCOOH  Mass production rate  (g h^-1^ cm^-2^) | Power density  (W cm^-2^) | Electricity consumption  (kWh kg^-1^ HCOOH) |
| --- | --- | --- | --- | --- | --- | --- | --- |
| 50 | 25.00 ± 3.59 | 50.01 ± 7.18 | 1.80 | 0.466 ± 0.067 | 0.0215 ± 0.0031 | 0.090 | 4.19 ± 0.60 |
| 100 | 92.82 ± 2.89 | 92.82 ± 2.89 | 2.12 | 1.732 ± 0.054 | 0.0797 ± 0.0025 | 0.212 | 2.66 ± 0.08 |
| 200 | 173.97 ± 5.15 | 86.99 ± 2.58 | 4.50 | 3.246 ± 0.096 | 0.1494 ± 0.0044 | 0.900 | 6.02 ± 0.18 |
| 300 | 252.71 ± 14.15 | 84.24 ± 4.72 | 6.20 | 4.715 ± 0.264 | 0.2170 ± 0.0122 | 1.860 | 8.57 ± 0.48 |
| 400 | 336.64 ± 12.60 | 84.16 ± 3.15 | 7.40 | 6.280 ± 0.235 | 0.2890 ± 0.0108 | 2.960 | 10.24 ± 0.38 |
| 500 | 231.60 ± 20.59 | 46.32 ± 4.12 | 8.60 | 4.321 ± 0.384 | 0.1989 ± 0.0177 | 4.300 | 21.62 ± 1.92 |
| 600 | 203.07 ± 23.46 | 33.85 ± 3.91 | 9.80 | 3.788 ± 0.438 | 0.1744 ± 0.0201 | 5.880 | 33.72 ± 3.90 |
| 700 | 161.92 ± 42.27 | 23.13 ± 6.08 | 10.70 | 3.021 ± 0.789 | 0.1390 ± 0.0363 | 7.490 | 53.87 ± 14.05 |

Note: Data are presented as mean ± standard deviation from n=3 independent electrolysis measurements.

**Table S4 The cathodic potentials corresponding to the applied current densities of Sn/SnO_x_-4@CB**

| Current density  (mA cm^-2^) | Cathodic potential  (V vs Ag/AgCl) | Cathodic potential  (V vs RHE) |
| --- | --- | --- |
| 50 | -1.571 | -1.256 |
| 100 | -1.994 | -1.679 |
| 200 | -2.431 | -2.116 |
| 300 | -2.745 | -2.430 |
| 400 | -2.982 | -2.667 |
| 500 | -3.210 | -2.895 |
| 600 | -3.430 | -3.115 |
| 700 | -3.660 | -3.345 |

Note: The potentials were measured against a saturated KCl Ag/AgCl reference electrode in 0.5 M K_2_SO_4_ electrolyte adjusted to pH 2. The reported cathodic potentials were not corrected for uncompensated resistance and may therefore include the ohmic potential drop between the cathode and the reference-electrode position.

**Table S5 ICP‑MS test results of Sn element in electrolyte before and after CO_2_RR electrolysis**

| Samples | Constant-volume  (mL) | Test element | Dilution factor | Sample elemental content (ppm) |
| --- | --- | --- | --- | --- |
| K_2_SO_4_^[a]^ | 50 | Sn | 10 | 0.0071 |
| K_2_SO_4_^[b]^ | 50 | Sn | 10 | 0.1083 |
| Note: The content of Sn element in the electrolyte solution before and after the test reaction  [a]before CO_2_RR; [b]after CO_2_RR | | | | |

**Table S6 Optimized structures of *COOH, OCHO, and H on the Li-, Na-, K-, and Cs-containing Sn/C models.**

| **Alkali metal cation** | ***COOH** | ***OCHO** | ***H** |
| --- | --- | --- | --- |
| **Li** |  |  |  |
|  |  |  |  |
| **Na** |  |  |  |
|  |  |  |  |
| **K** |  |  |  |
|  |  |  |  |
| **Cs** |  |  |  |
|  |  |  |  |

### **Table S7. Calculated free energies of the relevant states on the bare and alkali-metal-containing Sn/C models**

| Model | G(HCOOH) | G(*COOH) | G(*OCHO) | G(*H) | G(H_2_) |
| --- | --- | --- | --- | --- | --- |
| Bare Sn/C | 0.613 | 1.290 | 0.540 | 1.010 | 0.713 |
| Li-containing Sn/C | 0.613 | -0.521 | -0.613 | -0.203 | 0.448 |
| Na-containing Sn/C | 0.613 | -0.462 | -0.611 | -0.323 | 0.479 |
| K-containing Sn/C | 0.613 | -0.922 | -1.047 | -0.405 | 0.675 |
| Cs-containing Sn/C | 0.613 | -0.757 | -0.857 | -0.357 | 0.614 |

**Table S8. Experimental and derived parameters used in the assessment**

| **Parameter** | **Symbol** | **Value** | **Unit** | **Data type/source** |
| --- | --- | --- | --- | --- |
| Functional unit | $\text{FU}$ | $\text{1}$ | kg pure HCOOH | Defined for LCA and TEA |
| Total current density | $\text{j}_{\text{total}}$ | $\text{400}$ | mA cm^-2^ | Experimental |
| HCOOH partial current density | $\text{j}_{\text{HCOOH}}$ | $\text{336.64}$ | mA cm^-2^ | Experimental, Table S3 |
| HCOOH Faradaic efficiency | $\text{F}\text{E}_{\text{HCOOH}}$ | $\text{84.16}$ | % | Calculated from $\text{j}_{\text{HCOOH}}\text{/}\text{j}_{\text{total}}$ |
| Full-cell voltage | $\text{V}_{\text{cell}}$ | $\text{7.4}$ | V | Experimental at 400 mA cm^-2^ |
| Demonstrated operating time | $\text{t}_{\text{stable}}$ | $\text{18}$ | h | Experimental |
| Geometric electrode area | $\text{A}_{\text{lab}}$ | $\text{1}$ | cm² | Experimental |
| Catalyst loading | $\text{L}_{\text{cat}}$ | $\text{0.75}$ | mg cm^-2^ | Experimental |
| HCOOH areal production rate | $\text{r}_{\text{HCOOH}}$ | $\text{0.289}$ | g cm^-2^ h^-1^ | Experimental, Table S3 |
| HCOOH areal production rate | $\text{r}_{\text{HCOOH}}$ | $\text{2.89}$ | kg m^-2^ h^-1^ | Unit conversion |
| Electrolyzer-level electricity demand | $\text{E}_{\text{elec}}$ | $\text{10.24}$ | kWh kg^-1^ HCOOH | Experimental/derived, Table S3 |
| CO_2_ feed rate | $\text{F}_{\text{CO}_{\text{2}}}$ | $\text{30}$ | sccm | Experimental base condition |
| Calculated single-pass carbon efficiency | $\text{SPCE}$ | $\text{8.5}$ | % | Calculated from CO_2_ feed and HCOOH production rate |
| Catholyte | — | 0.5 M K_2_SO_4_, pH 2 | — | Experimental |
| pH-adjusting reagent | — | H_2_SO₄ | — | Experimental |
| Membrane | — | Proton-exchange membrane | — | Experimental |
| Anode | — | Pt plate | — | Experimental |
| Gas-diffusion substrate | — | Carbon paper | — | Experimental |
| Hydrophobic agent | — | PTFE | — | Experimental |
| PTFE loading on the gas-facing side | $\text{L}_{\text{PTFE}}$ | $\text{0.75}$ | mg cm^-2^ | Experimental |
| Catalyst-layer binder | — | Nafion | — | Experimental |
| Electron-transfer number | $\text{z}$ | $\text{2}$ | — | Reaction stoichiometry |
| Faraday constant | $\text{F}$ | $\text{96485}$ | C mol^-1^ | Physical constant |
| Molecular weight of HCOOH | $\text{M}_{\text{HCOOH}}$ | $\text{46.03}$ | g mol^-1^ | Chemical constant |
| Molecular weight of CO_2_ | $\text{M}_{\text{CO}_{\text{2}}}$ | $\text{44.01}$ | g mol^-1^ | Chemical constant |
| Theoretical electrical charge | $\text{Q}_{\text{th}}$ | $\text{1164}$ | Ah kg^-1^HCOOH | Derived from Faraday’s law |
| Stoichiometric CO_2_ demand | $\text{m}_{\text{CO}_{\text{2}}\text{,stoich}}$ | $\text{0.956}$ | kg CO_2_ kg^-1^HCOOH | Derived from reaction stoichiometry |
| Catalyst allocation over 18 h | $\text{m}_{\text{cat}}$ | $\text{0.144}$ | g catalyst kg^-1^HCOOH | Calculated from loading, productivity, and operating time |

**Notes:** The HCOOH Faradaic efficiency was calculated as 336.64/400=84.16%. The SPCE was calculated for a 1 cm² electrode at 400 mA cm^-2^ and a CO_2_ flow rate of 30 sccm, using a gas molar volume of 24.45 L mol^-1^ at 25 °C and 1 atm. The catalyst allocation assumes complete catalyst replacement after the demonstrated 18 h operating period and is therefore conservative.

**Table S9. Base-case assumptions and sensitivity ranges for the prospective life-cycle assessment**

| Parameter | Symbol | Base-case value | Sensitivity range | Unit | Basis |
| --- | --- | --- | --- | --- | --- |
| Functional unit | $\text{FU}$ | $\text{1}$ | Fixed | kg pure HCOOH | Defined |
| Grid electricity carbon intensity | $\text{E}\text{F}_{\text{grid}}$ | $\text{0.55}$ | $\text{0.03}$-$\text{0.65}$ | kg CO_2_e kWh^-1^ | Scenario assumption |
| Low-carbon electricity intensity | $\text{E}\text{F}_{\text{low−C}}$ | $\text{0.03}$ | $\text{0.02}$-$\text{0.05}$ | kg CO_2_e kWh^-1^ | Scenario assumption |
| CO_2_ supply emission factor | $\text{E}\text{F}_{\text{CO}_{\text{2}}}$ | $\text{0.20}$ | $\text{0.05}$-$\text{1.00}$ | kg CO_2_e kg^-1^ CO_2_ | Includes capture, purification, and compression |
| Auxiliary electricity fraction | $\text{f}_{\text{aux}}$ | $\text{10}$ | $\text{5}$-$\text{15}$ | % of electrolysis electricity | Pumps, circulation, temperature control, and gas handling |
| Auxiliary electricity demand | $\text{E}_{\text{aux}}$ | $\text{1.024}$ | $\text{0.512}$-$\text{1.536}$ | kWh kg^-1^ HCOOH | Calculated from $\text{f}_{\text{aux}}\text{×}\text{E}_{\text{elec}}$ |
| Product-separation energy | $\text{E}_{\text{sep}}$ | $\text{5.0}$ | $\text{0.5}$-$\text{8.0}$ | kWh kg^-1^ HCOOH | Screening assumption |
| Total electricity demand | $\text{E}_{\text{total}}$ | $\text{16.264}$ | Scenario dependent | kWh kg^-1^ HCOOH | Electrolysis, auxiliary operation, and separation |
| Fresh CO_2_ demand with recycling | $\text{m}_{\text{CO}_{\text{2}}\text{,fresh}}$ | $\text{0.956}$ | Fixed by stoichiometry | kg CO_2_ kg^-1^ HCOOH | Base case |
| CO_2_ input without recycling | $\text{m}_{\text{CO}_{\text{2}}\text{,no−rec}}$ | $\text{11.25}$ | SPCE dependent | kg CO_2_ kg^-1^ HCOOH | Calculated using an SPCE of 8.5% |
| CO_2_ recycling | — | Yes | Yes/No | — | Base case assumes recycling |
| Catalyst synthesis emission factor | $\text{E}\text{F}_{\text{cat}}$ | $\text{50}$ | $\text{10}$-$\text{100}$ | kg CO_2_e kg^-1^ catalyst | Screening assumption |
| Catalyst allocation period | $\text{t}_{\text{cat}}$ | $\text{18}$ | $\text{18}$-$\text{1000}$ | h | Experimental base case and prospective range |
| Catalyst use per functional unit | $\text{m}_{\text{cat}}$ | $\text{0.000144}$ | Lifetime dependent | kg catalyst kg^-1^ HCOOH | Derived |
| CO_2_ incorporated into HCOOH | $\text{m}_{\text{CO}_{\text{2}}\text{,embedded}}$ | $\text{0.956}$ | Fixed | kg CO_2_ kg^-1^ HCOOH | Reported separately |
| Time horizon for GWP | — | $\text{100}$ | Fixed | yr | GWP100 |
| Permanent sequestration credit | — | Not applied | — | — | HCOOH use may release the incorporated carbon |
| Electrolyzer hardware manufacturing | — | Excluded | — | — | Insufficient material and lifetime inventory |
| Pt-anode manufacturing burden | — | Not separately quantified | — | — | Included as a limitation |
| Detailed solvent inventory | — | Not separately quantified | — | — | Separation represented by an aggregate energy term |

**Notes:** The base-case boundary is cradle-to-gate and includes catalyst and electrode preparation, CO_2_ supply and recycling, electrolysis, auxiliary electricity, product recovery, electrolyte handling, and waste treatment. The HCOOH outlet concentration and detailed separation conditions were not measured

**Table S10. Base-case assumptions and sensitivity ranges for the screening-level techno-economic assessment**

| Parameter | Symbol | Base-case value | Sensitivity range | Unit | Basis |
| --- | --- | --- | --- | --- | --- |
| Nominal plant capacity | $\text{P}_{\text{annual}}$ | $\text{10,000}$ | $\text{1,000}$-$\text{100,000}$ | $\text{tHCOOH}\text{ }\text{yr}^{\text{-}\text{1}}$ | Prospective plant scale |
| Capacity factor | $\text{CF}$ | $\text{90}$ | $\text{70}$-$\text{95}$ |  | Assumption |
| Annual operating time | $\text{H}$ | $\text{7,884}$ | $\text{6,132}$-$\text{8,322}$ | $\text{h}\text{yr}^{\text{-}\text{1}}$ | Calculated from capacity factor |
| Electricity price | $\text{p}_{\text{elec}}$ | $\text{0.05}$ | $\text{0.02}$-$\text{0.10}$ | $\text{USD}\text{ }\text{kWh}^{\text{-}\text{1}}$ | Base case |
| Low-carbon electricity price | $\text{p}_{\text{low−C}}$ | $\text{0.03}$ | $\text{0.02}$-$\text{0.06}$ | $\text{USD}\text{ }\text{kWh}^{\text{-}\text{1}}$ | Scenario assumption |
| $\text{CO}_{\text{2}}$ price | $\text{p}_{\text{CO}_{\text{2}}}$ | $\text{50}$ | $\text{20}$-$\text{150}$ | $\text{USD}\text{ }\text{t}^{\text{-}\text{1}}\text{CO}_{\text{2}}$ | Captured and purified $\text{CO}_{\text{2}}$ |
| Catalyst price | $\text{p}_{\text{cat}}$ | $\text{100}$ | $\text{20}$-$\text{500}$ | $\text{USD}{\text{ }\text{kg}}^{\text{-}\text{1}}\text{catalyst}$ | Screening assumption |
| Installed electrolyzer cost | $\text{C}_{\text{stack}}$ | $\text{3,000}$ | $\text{1,000}$-$\text{10,000}$ | $\text{USD}{\text{ }\text{m}}^{\text{-}\text{2}}$ | Includes stack and basic auxiliaries |
| Membrane price | $\text{p}_{\text{mem}}$ | $\text{500}$ | $\text{200}$-$\text{1,000}$ | $\text{USD}\text{ }\text{m}^{\text{-}\text{2}}$ | Screening assumption |
| Membrane lifetime | $\text{t}_{\text{mem}}$ | $\text{1}$ | $\text{0.5}$-$\text{5}$ | $\text{yr}$ | Scenario assumption |
| GDE price | $\text{p}_{\text{GDE}}$ | $\text{100}$ | $\text{50}$-$\text{500}$ | $\text{USD}\text{ }\text{m}^{\text{-}\text{2}}$ | Carbon paper and hydrophobic treatment |
| GDE lifetime | $\text{t}_{\text{GDE}}$ | $\text{1}$ | $\text{0.5}$-$\text{5}$ | $\text{yr}$ | Scenario assumption |
| Catalyst lifetime | $\text{t}_{\text{cat}}$ | $\text{18}$ | $\text{18}$-$\text{1,000}$ | $\text{h}$ | Demonstrated base case and prospective range |
| Discount rate | $\text{i}$ | $\text{8}$ | $\text{5}$-$\text{12}$ |  | Financial assumption |
| Plant lifetime | $\text{N}$ | $\text{15}$ | $\text{10}$-$\text{25}$ | $\text{yr}$ | Financial assumption |
| Capital recovery factor | $\text{CRF}$ | $\text{0.1168}$ | Scenario dependent | — | Calculated from $\text{i}$ and $\text{N}$ |
| Fixed O&M fraction | $f_{O\&M}$ | $\text{4}$ | $\text{2}$-$\text{6}$ |  | Assumption |
| Electrolyte, water, and waste-treatment cost | $\text{C}_{\text{liquid}}$ | $\text{0.015}$ | $\text{0.005}$-$\text{0.050}$ | $\text{USD}\text{ }\text{kg}^{\text{-}\text{1}}\text{HCOOH}$ | Screening assumption |
| By-product $\text{H}_{\text{2}}$ credit | $\text{C}_{\text{H}_{\text{2}}}$ | $\text{0}$ | Not evaluated | $\text{USD}{\text{ }\text{kg}}^{\text{-}\text{1}}\text{HCOOH}$ | Excluded from base case |
| Taxes and profit margin | — | Excluded | — | — | Screening production-cost calculation |
| Financing during construction | — | Excluded | — | — | Not available |
| Required electrochemical area | $\text{A}_{\text{elec}}$ | $\text{438.9}$ | Performance dependent | $\text{m}^{\text{2}}$ | Calculated at $\text{10,000t}\text{yr}^{\text{−1}}$ |
| Installed stack CAPEX | $\text{CAPE}\text{X}_{\text{stack}}$ | $\text{1.317}$ | Performance dependent | $\text{million}\text{ }\text{USD}$ | Calculated from area and installed cost |

**Notes:** The calculated value is reported as a screening-level production cost rather than a bankable minimum selling price. Taxes, financing during construction, profit margins, and by-product credits were excluded. The Pt anode, flow fields, seals, and basic balance-of-plant components were represented through the installed electrolyzer cost.
